# Syndirella: synthesis-directed fragment elaboration enables extensive and cost-effective binding site exploration beyond catalog compounds

**DOI:** 10.1039/d6sc02032a

**Published:** 2026-09-29

**Authors:** Kate K. Fieseler, Max Winokan, Joseph A. Morrone, Charlotte M. Deane, Frank von Delft, Warren Thompson

**Affiliations:** a Oxford Protein Informatics Group, Department of Statistics, University of Oxford Oxford OX1 3LB UK; b Research Complex at Harwell Harwell Science and Innovation Campus Didcot OX11 0FA UK; c Centre for Medicines Discovery, University of Oxford Oxford OX3 7DQ UK; d Diamond Light Source Harwell Science and Innovation Campus Didcot OX11 0QX UK warren.thompson@diamond.ac.uk; e IBM TJ Watson Research Center, IBM Research 1101 Kitchawan Rd, Yorktown Heights NY 10598 USA; f Department of Biochemistry, University of Johannesburg Auckland Park Johannesburg 2006 South Africa

## Abstract

Fragment screens provide an information-rich starting point for designing derivative compounds in structure-based drug discovery. However, derivatives in the fragment-to-hit stage are typically designed from only one or a few fragments, risking premature convergence on an unproductive series and dramatically limiting exploration of the binding site's interaction opportunities. This is avoidable by exploring a broad, chemically diverse set of scaffolds and elaborations spanning interactions revealed across many fragments, as well as those not yet observed. Conventional catalog procurement makes this breadth of testing prohibitively expensive for early discovery. In-house high-throughput (HT) synthesis can raise interaction productivity, the number of unique predicted protein–ligand interactions achievable within a given budget, by lowering the cost per compound, including labor and consumables, yet no design tool exists for this purpose. We introduce *Syndirella* (Synthesis Directed Elaborations), which presents a candidate elaboration space around input fragment-derived scaffolds, constrained to HT-compatible reactions and catalog reactants, for cost-effective sampling of many scaffolds' elaborations in a single experimental cycle. Applied to three viral protein targets, Syndirella designs demonstrated pharmacophore diversity, recapitulating known fragment interactions, identifying novel functionally relevant ones, and accessing chemical space absent from commercial catalogs. A companion paper details validation on a calcium-binding protein, yielding crystallographic structures and measurable binding, with in-house synthesis achieving a 71% cost reduction over catalog purchasing, labor and consumables included. Reactant rather than product procurement gives potentially 2.5 times as many accessible compounds and higher interaction productivity for the same budget. Syndirella is most impactful for structure-enabled campaigns with fragment-bound crystal structures, robust HT chemistry, and assays compatible with crude reaction mixtures, and is available at https://github.com/oxpig/syndirella.

## Abstract 2

Syndirella proposes elaborations of fragment-derived scaffolds for high-throughput synthesis using in-catalog reactants for SAR exploration.

## Graphical abstract

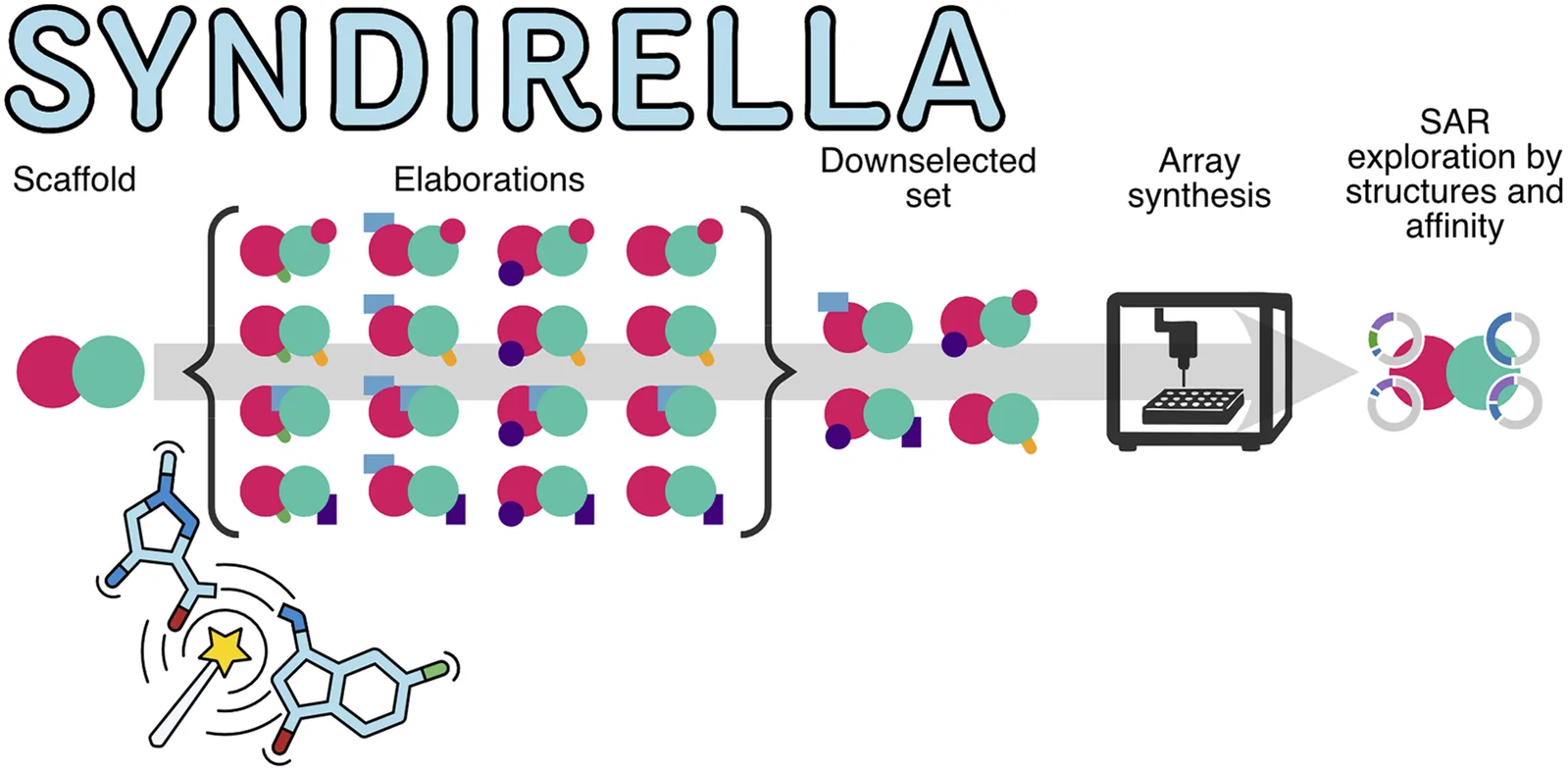

## Introduction

Fragment-based drug discovery (FBDD) has established itself as an effective method that provides starting points for compound design towards desired activity against the function of a protein.^1,2^ It operates under the assumption that, in the early hit-to-lead stage, small molecules (<250 Da) are more informative than the larger molecules typical of traditional high-throughput (HT) screening.^2,3^ Fragment screens usually have a higher hit rate, a higher percentage of interacting atoms (higher ligand efficiency), and more effective sampling of chemical space.^4,5^ Structural methods can resolve fragments' conformations and interactions, and their simplicity leaves room for development through simple chemistry. This informative fragment dataset provides the foundation for knowledge-based compound design; however, fully utilizing structural information from the whole fragment screen remains challenging and usually only one or a few fragments are considered in designing derivatives.^6,7^ This is because there are no systematic approaches for routinely advancing information from many fragments to compounds with measurable potency suitable for lead optimization.

Drug discovery often relies on iterative Design-Make-Test-Analyze (DMTA) cycles that, while systematic, suffer from fundamental limitations in their ability to always find optimal solutions to the drug discovery problem.^8^ These sequential approaches do not guarantee convergence to the best possible compound, as they are inherently susceptible to becoming trapped in local solution minima. Each DMTA iteration typically explores only a small subset of chemical modifications around a limited number of the most promising compounds, creating a step-wise optimization process that may miss superior solutions located in distant regions of chemical space.^9^ This incremental approach can lead to suboptimal drug candidates, as the final compound is heavily dependent on the initial starting points and the specific pathway taken through chemical space, rather than representing a true global optimum.

The obvious places to start to implement a more comprehensive exploration strategy for fragment progression are the Design and Make components of the DMTA cycle. For the design component, one of the early priorities with fragment progression is to increase the affinity of the compound by increasing the number of non-covalent interactions with relevant residues.^10–12^ The occurrence of these productive interactions depends on multiple factors, including appropriate geometric alignment between ligand and protein atoms, conformational accessibility of the bound state, and various experimental conditions.^13^ One systematic method for fragment progression is to explore structure–activity relationships (SAR) of fragments or fragment-inspired designs through congeneric series—sets of compounds that share a common core structure but differ in their substituents.^2,14,15^ Although navigating the possible design space is not facile, as the estimated SAR space around a fragment up to 26 heavy atoms encompasses approximately 10^13^ compounds, many of which lack straightforward synthetic routes.^16^ If the earliest designs require expensive resourcing, the amount of sampling will be affected with fewer designs tested and the accessible chemical space reduced.^17,18^ Exploring such vast SAR space requires a fundamental shift in the scale of experiments, moving from traditional single-compound synthesis and testing to massively parallel approaches that can handle hundreds to thousands of compounds simultaneously.^19–25^

To maximally support this massive SAR exploration, high-throughput (HT) micro- and nano-scale chemistry that produces hundreds to thousands of crude reaction mixtures (CRMs),^26–30^ combined with HT X-ray crystallography based binding site-directed purification strategies,^31–34^ can be coupled to maximally explore the fragment progression chemical space. This direct-to-biology approach leverages low-cost liquid handling platforms to perform plate-based synthesis while eliminating the need for conventional purification steps. Instead of isolating individual compounds through traditional chromatographic methods, the protein target's binding site serves as a molecular filter, selectively enriching active compounds directly from crude mixtures through affinity-based interactions determined by X-ray crystallography. Critically, because X-ray crystallography resolves the electron density of the bound molecule itself, it provides direct structural proof of which component of a mixture is bound, removing ambiguity as to whether observed binding reflects the desired ligand or a byproduct or reagent in the CRM.

This binding site purification strategy, introduced by Grosjean *et al.*,^31^ enables the evaluation of thousands of CRMs, where only those with meaningful target affinity are retained and characterized. This integrated approach offers significant advantages over traditional compound procurement and purification workflows. While conventional methods require extensive purification of each individual compound before biological testing, imposing substantial costs and extended timelines that slow iterative DMTA cycles, the binding site purification approach has the potential to bypass these bottlenecks.

Recent chemistry advances have significantly improved the feasibility of implementing liquid-handling platforms for HT micro- and nano-scale chemistry, expanding both synthetic tractability and the variety of accessible pharmacophoric space.^35^ These advances include the development of catalysts for ambient conditions, such as G3 and G4 Buchwald palladium precatalysts that enable diverse cross-coupling reactions^36,37^ and innovative reactor designs for high-throughput electrochemistry.^38^ Particularly promising are novel approaches for multi-step library synthesis in continuous flow that incorporate multiple synthetic methodologies, as demonstrated by Pijper *et al.* who developed an assembly line approach for generating diverse compounds through multi-vectorial exploration.^39^ These advances make routine use of HT chemistry possible in DMTA cycles, although there has been little leverage by computer-aided drug design (CADD) tools.

While the experimental proof-of-principle for HT synthesis and testing CRMs has been shown, there are no CADD tools made to output compound sets specifically to be synthesized *via* these platforms for FBDD. Current approaches that present a combinatorially produced chemical space by reaction based enumeration such as V-SYNTHES and tools offered by BioSolveIT show experimental success but have been used in the classical approach of presenting a compound set that has been heavily filtered to a manageable set for purchase from make-on-demand catalogs (usually <90 compounds).^40–43^ While successful in their context, if applied to produce compound sets designed to be synthesized in-house by liquid handling robotics, there are missed opportunities for economies of scale through shared synthetic routes and starting materials.^44–46^ Furthermore, existing reaction based enumeration typically focus on single-step transformations or limited reaction sets, constraining the discovery space. The feasibility of routinely designing and performing multi-step synthesis has become more attainable from the advancements of computer aided synthesis planning tools such as AiZynthFinder,^47,48^ ASKCOS,^49^ and Manifold (https://postera.ai/) which allow for more reliable algorithmic proposal especially for common reactions. There is a clear need for CADD tools that can generate large, diverse libraries of synthetically tractable compounds specifically designed for HT synthesis platforms, while maximizing the exploration of the SAR chemical space around fragment hits.

Here, we introduce a new tool Syndirella (Synthesis Directed Elaborations) that presents a candidate elaboration space around a fragment derived scaffold constrained to a set of HT-compatible reactions and to reactants available in catalogs, and interfaces with external tools for synthesis planning and pose prediction. Syndirella enables testing 100s–1000s of elaborations across scaffolds, with users able to encode their own chemistry, modify retrosynthetic routes, and select cost-effective reactants for in-house synthesis. By lowering the cost per compound in this way, Syndirella allows experiments with high interaction productivity, the number of unique predicted protein–ligand interactions achievable within a given budget.

We applied Syndirella to three protein targets, demonstrating structural SAR exploration and success with a greater breadth-to-depth ratio than traditional catalog superstructure search methods by leveraging reactant-based procurement to generate novel elaborations absent from commercial catalogs. These catalog-absent compounds exhibit high predicted interaction productivity, extensively exploring the binding site and both recapitulating and identifying novel, key residue interactions relative to the original fragment screen. Syndirella was also applied experimentally to a fragment screen of a calcium-binding protein, validating its usefulness for SAR elucidation with structural and kinetic readout, detailed in a companion paper by Muñoz-Reyes *et al.*^50^

The approach is most impactful for structure-enabled FBDD campaigns with fragment-bound crystal structures, robust HT chemistry, and assay formats compatible with CRMs.

## Methods

Syndirella takes as input fragment or fragment-derived scaffold structures and outputs congeneric series with 3D poses and associated synthetic routes suitable for HT synthesis. For each scaffold, synthetic routes are first solved using a retrosynthesis tool or defined manually, and for each required reactant, a superstructure search is performed under constraints that preserve the reaction site. The resulting reactant superstructures are combined to yield a large set of elaborated products, which are then energy-minimized *in silico* into the experimental protein structure with strong constraints to the experimental fragment pose using Fragmenstein.^51^ Predicted conformations are subsequently scored and filtered based on geometry checks and modeled binding energy. The pipeline is tunable and expandable to accommodate the specific capabilities and preferences of downstream HT synthesis experiments.

### Computational workflow

Syndirella produces congeneric series of compounds from input scaffolds with fragment origin. These scaffolds can be fragments themselves or derivatives from fragment growing, linking, or most commonly in this work, fragment merges.^52^ Fragment merged compounds can be proposed algorithmically or manually, although design methods should prioritize the recapitulation of fragment atoms. One algorithmic method that implements fragment merging and is extensively used throughout our pipeline is Fragmenstein.^51^ Fragmenstein combines atoms from fragments using structural information to create derivatives that are predicted to adopt the same binding pose as the fragments. Additionally, Wills *et al.* utilized the Fragment Network,^53,54^ first described by Astex Pharmaceuticals,^15^ where traversing a graph database of compounds starting from a fragment node leads to derivative compounds that recapitulate fragment–protein interactions. Compounds that are used as input into the complete Syndirella workflow are hereafter referred to as scaffolds, shown in Fig. 1.

**Fig. 1:**
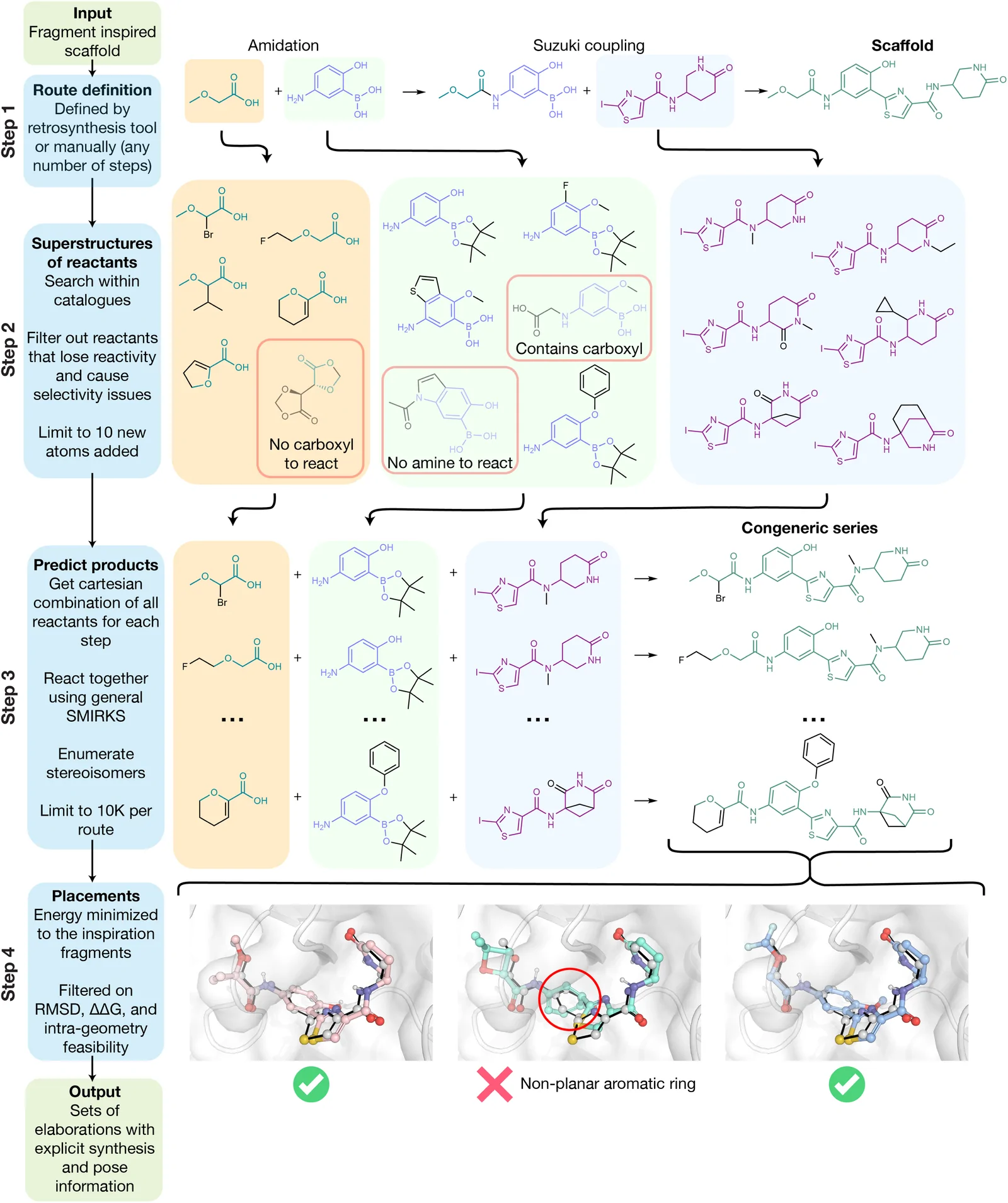
Workflow of Syndirella starting from a fragment-inspired scaffold, the pipeline defines synthetic routes, identifies superstructures of reactants from chemical catalogs, predicts products *via* combinatorial synthesis, and performs placement relative to inspiration fragments using Fragmenstein.^51^ The example shows a two-step synthesis (amidation and Suzuki coupling) where final products (thick representation) are overlaid with original fragments (thin representation), with successful placements indicated by green checkmarks and not successful by red crosses.

### Pose generation of scaffolds and filtering

First, poses are generated for input scaffolds to filter out those which do not have an energetically favorable conformation in the binding site. Pose generation is performed using the tool Fragmenstein,^51^ although any pose generation method could be used, whether restrained to the parent fragment pose or not. Fragmenstein generates an initial pose by calculating the positional overlap between the compound and fragments used for the original merge. The atoms on the compound found in this overlap are restricted to the coordinates from the fragments. This unphysical conformation undergoes energy minimization placed in the apo protein template specified by the user. Energy minimization is controlled by either PyRosetta's scorefunction ref2015 (ref. 55) or RDKit's UFF.^56^ For the analysis shown here, minimization with RDKit's UFF was performed unless specified otherwise. When using PyRosetta, the sidechains of protein residues with alpha carbons within 8 Å of the centroid of the compound are energy minimized whereas the protein is fixed when performing UFF minimization. A ΔΔ*G* score is calculated by Fragmenstein as a single snapshot difference of the final bound state minus the unbound state (see ref. 51). It should be mentioned that this ΔΔ*G* score should not be interpreted as a robust free binding energy and instead is used only as a filter where if the score is ≥0 the pose is most likely energetically unfavorable and strained.

Scaffolds that complete energy minimization within 240 s, have an RMSD of <2 Å to the inspiration fragments, have a ΔΔ*G* score of <0, and pass PoseBuster's intramolecular geometry checks for bond lengths, angles, steric clashes, and aromatic ring planarity are accepted.^57^

### Route modeling for HT synthesis

For each scaffold that passes pose generation, Syndirella proposes elaborations by explicit SMIRKS-encoded forward synthesis route modelling with any number of reaction steps.^58^ First, route selection is specified for the scaffold manually or proposed by a retrosynthesis tool. Here in this analysis using Manifold (https://postera.ai/), although functionality using the open-source tool AiZynthFinder is also provided. When using Manifold, the catalogs specified to query were enamine_bb, mcule, mcule_ultimate, enamine_real, enamine_made, and generic (common molecules that are found in most catalogs).^59–61^ For a compound to be considered “elaboratable”, there must be a forward synthesis route with all reactions encoded in Syndirella's SMIRKS library. Syndirella includes 109 SMIRKS encoded reactions which have been specifically chosen for HT chemistry. In this work 23 were used (available as RXN_SMIRKS_CONSTANTS.json on Zenodo^62^) and since this analysis, more have been added with a significant amount from Dobbelaere *et al.*^63^ and are found at https://github.com/oxpig/syndirella.

### Syndirella prioritizes user synthesis preferences

Syndirella's reaction repertoire can be expanded or edited by adding a SMIRKS pattern^58^ representing the reaction of interest (*e.g.*, sourced from resources such as SMARTS-RX^64^ or SmartReact^65^). Additionally, reaction substitutions can be specified to suit user preferences based on better yield history or usually cheaper reactants. For example, replacing Schotten–Baumann reactions with amidations often improves building block availability and reduces cost (Fig. S4). Each alternative reaction generates an additional and separate synthesis route and can be specified in a .json format and example jupyter notebooks to generate these files are provided (see https://github.com/oxpig/syndirella).

### Superstructure search of reactants to produce elaborated products

To produce the series, each reactant in the forward synthesis route is elaborated and the full Cartesian product of the reactants is computed *in silico*. The following steps are taken for each reaction step in the forward synthesis route. First, the original reactants are labeled with the specific reactant number in the SMIRKS of that reaction. Then, each reactant is used as a query in a superstructure search – performed by Manifold in this work, although functionality using the catalog search tool Arthor (https://arthor.docking.org/) is provided. A superstructure of a query compound is a compound that contains the query compound as a maximum common substructure. In this analysis, the catalogs queried were enamine_bb, mcule, mcule_ultimate, enamine_real, enamine_made, and generic (common molecules that are found in most catalogs).^59–61^

Superstructure reactants are filtered by removing duplicates, compounds containing non-abundant isotopes, compounds that don't match the reactant SMIRKS, and if the number of atom difference to the query reactant is <0 or >10 (although these thresholds can be changed to user preferences). If the step is a bimolecular reaction, reactants that contain both reacting groups are excluded to reduce selectivity issues.

Product compounds are generated from the full Cartesian product of the smallest 100 reactants for bimolecular reactions or by the smallest 10 K reactants for unimolecular reactions, thereby restricting the total number of products in each step to ≤10 K (although this threshold can be changed to user preferences). For each reactant combination, the product is determined by the original SMIRKS reaction pattern. Products are excluded if they cannot be sanitized by the RDKit.^66^ If multiple products are produced for a single combination, the product is flagged with a selectivity issue label.

The previous steps repeat for each reaction step until the final products are determined and labeled with their InChi key and ordered in ascending atom count. All stereoisomers are enumerated and the SMILES and full routes are stored as a dataframe.^67,68^

### Pose generation of products and filtering

Each compound must be placed into the protein pocket to predict the interactions between the compound and residues. The smallest 10 K elaborations after stereo enumeration are placed and filtered following the same steps detailed above for scaffold placement, although this threshold can be changed to user preferences.

### XChem data sets

The three test cases used in this work are publicly available crystallographic fragment screens from the XChem facility,^69,70^ summarized in Table 1 and are available for download from the Fragalysis platform (https://fragalysis.diamond.ac.uk). Scaffolds from these datasets were derived from three sources: computational fragment merging algorithms (either Knitwork^53,54^ or Fragmenstein^51^), exact fragment hits, or manually designed fragment merges.

**Table 1:** Summary of scaffolds used as test data

Target	Origin	Count	Unique fragment combinations
Chikungunya virus nsP3 macrodomain (CHIKV-Mac)	Fragmenstein	317	616
	Manual	14	
	**Total**	**331**	
Coxsackievirus A16 2A protease (EV-A71-2A)	Exact	3	48
	Knitwork	42	
	Manual	76	
	**Total**	**121**	
Enterovirus D68 3C protease (EV-D68-3C-PROA)	Knitwork	291	58
	**Total**	**291**	

The Chikungunya virus nsP3 macrodomain protein (CHIKV-Mac) performs removal of ADP-ribose modifications to host proteins, an essential part of the replication process for Chikungunya virus.^71^ It has been shown mutants affecting ADP–ribose binding slows viral replication in mammalian cells and reduces virulence.^72^ A fragment screen was performed, resulting in 66 fragments bound in the ADP–ribose binding site and 616 unique combinations were used to design scaffolds by Fragmenstein and Knitwork.^73^

Enterovirus A71 is a common cause of respiratory disease and hand, foot, and mouth disease in children worldwide.^74^ The 2A protease (EV-A71-2A) is responsible for a single intramolecular cleavage in the viral polyprotein, therefore targeting the active site could inhibit viral growth. Following a fragment screen on Coxsackievirus A16 2A protease which serves as a proxy for the highly conserved EV-A71-2A protein, 43 fragments were resolved in the active site and a variety of follow-up methods were utilized including manual designs to result in 121 scaffolds.^75^

Similar to enterovirus A71, enterovirus D68 results in respiratory illness and in severe cases, paralysis.^76^ The 3C protease (EV-D68-3C) is required to cleave the non-structural part of the viral polyprotein. EV-D68-3C also cleaves many host proteins to facilitate viral infection thus, inhibition can reduce the viral replication by this target. The fragment screen of this target resulted in 16 fragments resolved in the active site, and only the Knitwork tool was used to propose 291 scaffolds.^77^

All scaffolds input into the pipeline totaled to 743, hereafter referred to as the original-743. After inputting into Syndirella, successful series were defined as having >3 unique products and >0 successful placements of elaborations, totaling to 234, hereafter referred to as the successful-234 (Fig. S1).

### Cheminformatic analysis

Morgan fingerprints with a radius of three and bit length of 2048 were used for all fingerprinting calculations using the RDKit.^66,78^ For the per cluster analysis, the mean Tanimoto values were calculated by taking the row-wise mean across the full Tanimoto similarity matrix for each elaboration in the cluster against all other molecules. Histograms of the mean Tanimoto values per cluster and smoothed by kernel density estimation using Seaborn's default parameters were calculated.^79^ All displays of chemical space by two dimensions were carried out by calculating the 50 first components from principal component decomposition of the Morgan fingerprints then transforming those components using the t-SNE algorithm. These transformations were carried out using the default parameters in scikit-learn.^80^

R-group decomposition was carried out with the RDKit's RGroupDecompose function where the cores required to identify the R positions were the input scaffolds used.^66^ Then the R groups were filtered by removing repeats across all R positions and those with more than one attachment point. R-group pharmacophores were assigned per atom using the RDKit^66^ using the following custom rules. Hydrophobic, carbon with no N/O/S neighbour. Hydrogen bond donor (HBD), N or O bearing ≥1 hydrogen. Hydrogen bond acceptor (HBA), N or O bearing no hydrogen, or a neutral NH. Halogen, F/Cl/Br/I. Classes are non-exclusive. Each R group was summarized by the shortest topological (bond count) distance from the attachment point at which each class occurred, giving one pharmacophore-distance pair per class per R group.

For the missing chemistry analysis, the original-743 were queried by Postera's Manifold for the top five retrosynthesis routes ordered by Manifold using the same catalogues as in Syndirella's default pipeline specifications.

### Comparison to traditional superstructure methods

The scaffolds used as input for Arthor (https://arthor.docking.org/) and Manifold superstructure querying were a subset of the original-743 that passed placement filtered by the same constraints as described in the Computational workflow filtering section, resulting in 660 scaffolds, hereafter referred to as the scaffold-pass-660 (Fig. S1). For carrying out the Arthor queries, we queried the following databases: REAL-Database-22Q1, Mcule-22Q1-8.7M, Mcule-BB-22Q1-2.1M, Mcule-Full-22Q1-60M, Mcule-V-22Q1-51M, mcule_purchasable_virtual_230121, and Mcule-Ultimate-20Q2-126M. To reduce artificial attrition, a maximum of 10 K results per database query were analyzed (compared to 100 from the usual workflow). For the Manifold queries, we queried the following databases: enamine_bb, mcule, mcule_ultimate, enamine_real, enamine_made, and generic (common molecules that are found in most catalogs). For each database, a 900-page limit was applied to avoid unnecessary truncation (compared to 10 from the usual workflow). The compounds produced by Syndirella that were included in this analysis were all elaborations before filtering by placement. Compounds from all methods were compared by InChI key of the SMILES with all stereoisomer information removed.^81^

The databases queried were matched as closely as possible across all methods to Syndirella's default workflow (Enamine^61^ BB, REAL, and MADE, and Mcule^59^). All queries were run in early 2025 using the exact same SMILES of the scaffold-pass-660 with all stereochemistry information removed.

### Pricing analysis

Compounds selected for catalog querying were those that passed the placement filters detailed above, totaling 234 scaffolds and 180 885 elaborations (including scaffolds). From this set, 5 K compounds for each target were sampled randomly based on index. Then the reactants used in each of the products synthesis routes and products were sent in for an availability quote to Enamine^61^ in late 2024 for 1.2 µM DMSO stocks. For the reactants query, only searching the Enamine building block catalog was allowed. If all reactants were available, the product was marked as reactant purchasable.

### Prediction of protein–ligand interactions

Protein–ligand interactions were analyzed by the tool Hit Interaction Profiling for Progression Optimization (HIPPO, see https://github.com/mwinokan/HIPPO), unless noted otherwise. HIPPO identifies protein–ligand interactions using RDKit features and maximum distance thresholds to define hydrogen bonding (donor–acceptor, 3.5 Å), π-stacking (aromatic–aromatic, 6.0 Å), π-cation (aromatic–PosIonizable, 4.5 Å), electrostatic (NegIonizable–PosIonizable, 4.5 Å), and hydrophobic interactions (hydrophobe–hydrophobe, 4.5 Å). Unique interactions were defined as concatenation of the interaction type, residue name, residue number, and chain.

### Experimental validation

Syndirella was applied to design elaborations from a fragment screen of the neuronal calcium sensor-1 protein (NCS-1). Detailed experimental procedures and results are described in Muñoz-Reyes *et al.*^50^ To briefly summarize, twelve scaffolds were designed algorithmically by the tool Fragmenstein^51^ from 17 unique fragments. These scaffolds were chosen to elaborate based on preferences for specific target regulation and accessible synthesis routes. All twelve scaffolds were processed through Syndirella with single step routes, resulting in 18 931 compounds placed and 5335 passed our filters of an RMSD of <2 Å to the fragments and a ΔΔ*G* score of <0.

The 5335-compound candidate set is the output of Syndirella and the stages that follow were performed independently. From this set, 590 were selected with the interaction identification and multi-objective optimization strategy tool, HIPPO https://github.com/mwinokan/HIPPO. HIPPO selects sets of elaborations that optimize criteria such as maximizing number included, number of predicted interactions, balance of elaborations per scaffold, *etc.*, while constrained to a reactant budget. A total of 454 elaborations were then attempted for synthesis using XChem's digitized chemistry platform, Chemist Assisted Robotics (CAR, see https://github.com/Waztom/xchem-CAR), for automated liquid handling, resulting in 252 elaborations successfully synthesized, a 55.5% success rate. Synthesis confirmation was achieved through automated LCMS analysis using MSCheck.^82^ The crude reaction mixtures were screened by X-ray crystallography at XChem,^69,70^ resulting in 14 compounds resolved. Additionally, a grating coupled interferometry (GCI) assay was performed measuring kinetic off-rate values.

Enumeration and placement were performed by Syndirella, compound selection by HIPPO, and synthesis by CAR. The experimental outcomes reported here therefore reflect the combined pipeline rather than the contribution of any single component.

## Results

We tested the ability of Syndirella to propose elaborations around fragments and fragment merged scaffolds for three viral protein targets: CHIKV-Mac, EV-A71-2A, and EV-D68-3C-PROA. First, we assess the chemical and pharmacophore diversity of the elaboration series produced by Syndirella, resulting in binding site interaction exploration where elaborations recapitulate known fragment interactions and introduce novel interactions with functionally relevant residues. We then show how Syndirella can be used to make decisions on improving the experimental platform's repertoire of synthetic capabilities by identifying critical chemistry needed to expand series accessibility and the tunability of synthesis parameters. Through comparison with traditional superstructure search methods, we show how Syndirella accesses distinct regions of chemical space. Additionally we demonstrate that Syndirella enables a reactant-based purchasing strategy resulting in a broader chemical space exploration and higher predicted interaction productivity within typical budget constraints and outside of the catalog. Finally, the practical utility of the platform is validated through application to NCS-1, where over 400 Syndirella elaborations were accessible *via* reactant procurement compared with only two *via* direct product procurement. These elaborations were synthesized by HT in-house chemistry, resulting in resolved crystal structures and measurable binding kinetics for real-world SAR analysis.

### Syndirella produces congeneric series with pharmacophore diversity enabling binding site exploration

Syndirella produces diverse congeneric series—sets of compounds that share a common core structure but differ in their substituents—using explicit synthesis routes. Fig. 2 shows a representative result, where a single fragment-inspired scaffold yields a set of elaborations that share one synthesis route and vary in substituents and pharmacophores. For this CHIKV-Mac series, the scaffold is a fragment-merged compound and utilizes a single step Buchwald–Hartwig amination reaction to produce 5085 elaborated products (Fig. 2a). The series is pharmacophorically diverse as seen in Fig. 2b with examples of hydrogen bond acceptors, donors, halogens, and hydrophobic groups introduced at four unique R positions on the scaffold.

**Fig. 2:**
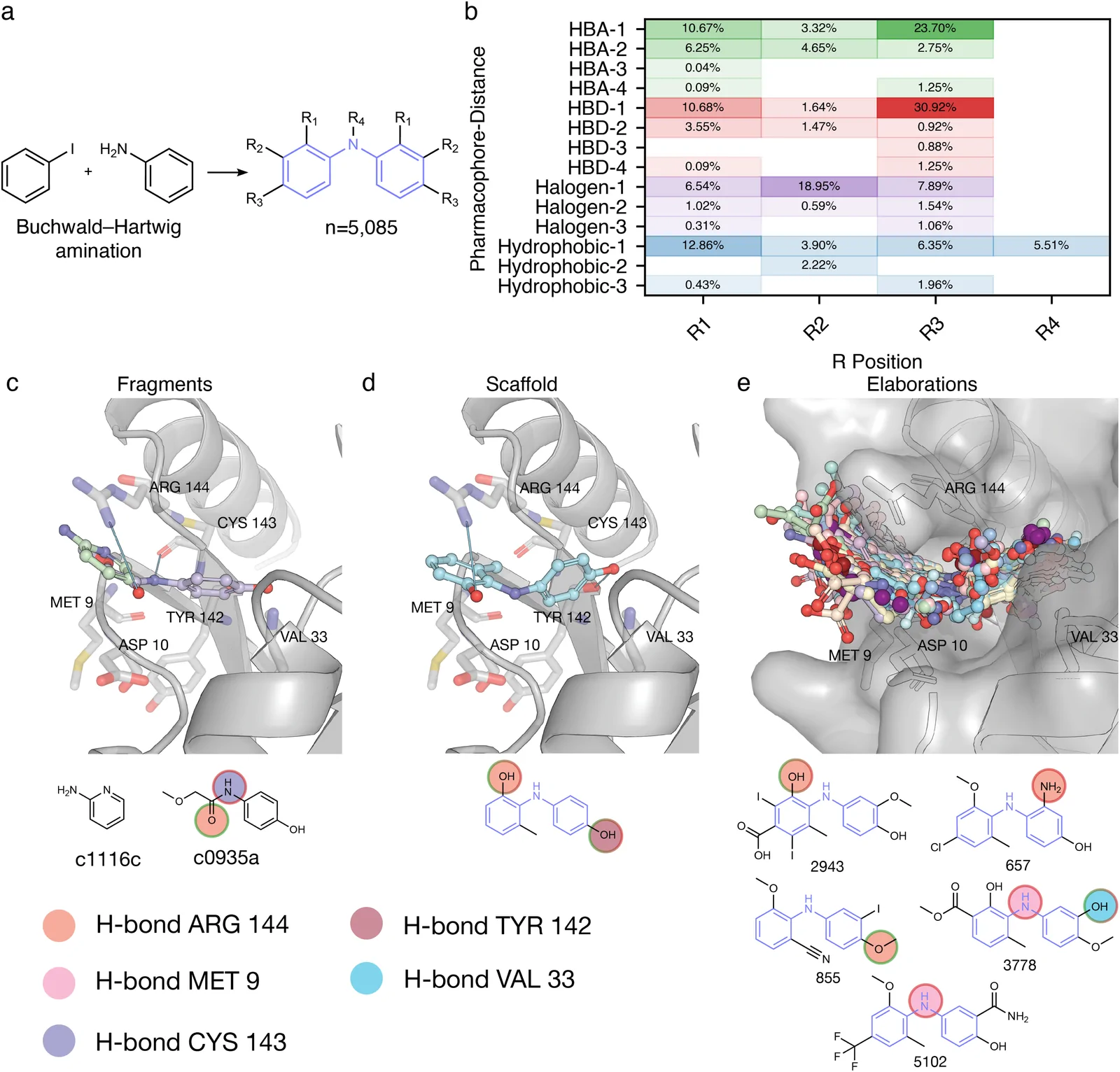
Syndirella produces a SAR series with pharmacophore diversity enabling binding site exploration. This series was designed for the CHIKV-Mac target. (a) The synthesis route (a single step Buchwald–Hartwig amination route) is shown for the scaffold and the four R positions from which the elaborations (*n* = 5085) vary on. (b) Heatmap of the pharmacophores introduced at the four R positions shown in (a), grouped into hydrogen bond acceptors (HBA), hydrogen bond donors (HBD), halogens, and hydrophobic groups and concatenated with their distance to the attachment point. The percent value shown is the percent of the pharmacophore-distance feature at each R position over the whole compound set. (c) The experimentally resolved structure of the inspiration fragments and hydrogen bond (H-bond) interactions identified by the protein–ligand interaction profiler^83^ shown by colored circles and labeled by their Fragalysis ID. Each H-bond interaction is outlined by the pharmacophore color matching the pharmacophore heatmap. (d) The predicted pose of the fragment merged scaffold calculated with Fragmenstein and predicted hydrogen bond interactions. (e) Fragmenstein predicted poses of 100 randomly sampled elaborations with five example elaborations shown in 2D, colored by the maximum common substructure with the scaffold, and predicted H-bond interactions shown.

The inspiration fragments resulted in two H-bond interactions experimentally resolved with ARG 144 and CYS 143, while the scaffold was predicted to interact with ARG 144 and TYR 142. Across the five example elaborations shown, additional predicted interactions emerge with MET 9 and VAL 33, demonstrating how the elaboration process can potentially expand the interaction profile beyond the original fragment hits.

Syndirella's systematic combinatorial enumeration achieves comparable pharmacophore coverage to early-stage experimental FBDD campaigns. In Fig. 3, the pharmacophore-distance heatmaps for Syndirella *versus* the COVID Moonshot series from the TRY-UNI-714a760b-6 scaffold show similar exploration patterns.^84^ The COVID Moonshot compounds represent initial follow-ups synthesized within the first four months since the start of the consortium, making them directly comparable to Syndirella's proposed first-iteration elaborations. While Syndirella's automated enumeration cannot match the full diversity of human and computer-designed compounds, it captures similar pharmacophoric positions prioritized in practice, demonstrating its utility as a tool for SAR exploration.

**Fig. 3:**
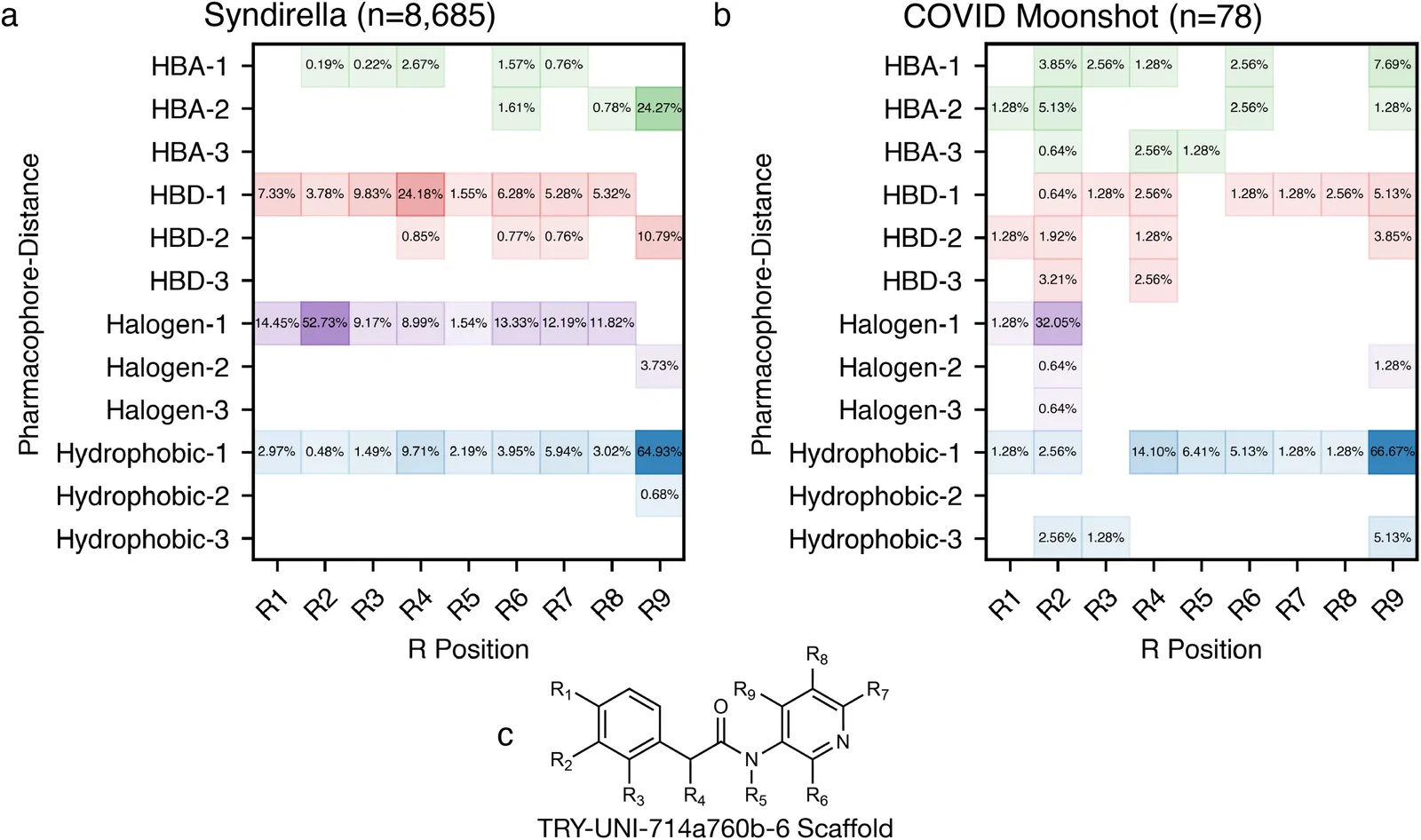
Syndirella's systematic approach is comparable with COVID Moonshot's heterogeneous design strategies in pharmacophore space. (a) Pharmacophore-distance heatmap for Syndirella-generated compounds (*n* = 8685) from the TRY-UNI-714a760b-6 scaffold, showing concentrated pharmacophore clustering at specific R positions. (b) Pharmacophore-distance heatmap for COVID Moonshot compounds (*n* = 78) from the 3-amino-pyridine like series made in the first four months since the start of the consortium (2020-03-17 to 2020-06-31). (c) Chemical structure of the TRY-UNI-714a760b-6 scaffold with R position labels (R1–R9). Heatmap intensity represents the percentage of compounds containing each pharmacophore type at each distance position. HBA = hydrogen bond acceptor, HBD = hydrogen bond donor.

For all series produced by Syndirella in this study, on average, a series contains over 61 unique R-groups after R-group decomposition (Fig. S2a). To provide another diversity metric of all series, the Tanimoto similarity distributions for CHIKV-Mac, EV-A71-2A, and EV-D68-3C-PROA are shown in Fig. S12–S19. Most demonstrate similar diversity profiles to the exemplar hit TRY-UNI-714a760b-6 (Fig. S11) where either the modes are similar to this series (∼0.2). While there are also less diverse series (mode > 0.4) these series usually have an order of magnitude less compounds than the 2 K present in the TRY-UNI exemplar series.

### Elaborations are predicted to explore new functionally relevant interactions not measured in the fragment screen

Syndirella elaborations are predicted to systematically explore the binding site's interaction landscape beyond what traditional fragment merging approaches can capture, recapitulating interactions with residues in the original crystallographic screen and identifying interactions with functionally relevant residues that were not measured. Seen in Fig. 4 the interaction exploration from the original fragment screen to fragment inspirations used for scaffold design and Syndirella generated elaborations is shown for CHIKV-Mac (Fig. 2). From the crystallographic fragment screen,^73^ there were 109 fragments resolved interacting with 73 unique residues. The predicted interactions for the sub-selected fragments for scaffold design (inspirations) and the scaffolds themselves is reduced compared to the fragment screen due to the subselection, but the frequency and diversity of predicted interactions increases dramatically with the elaborations, which are predicted to interact with 14 residues not found to interact in the original fragment screen (seen grouped in Fig. 4).

**Fig. 4:**
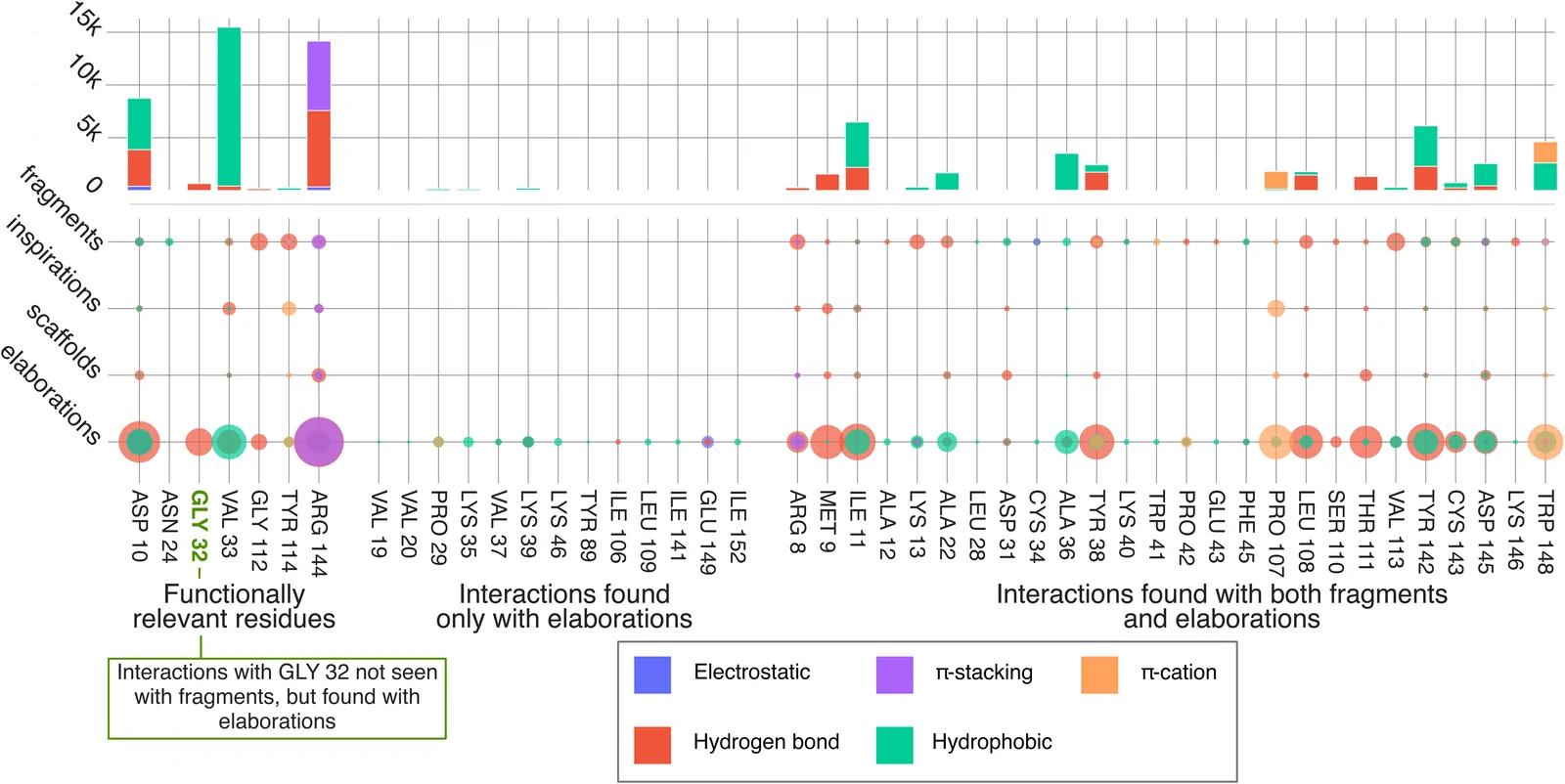
Syndirella elaborations explore both interactions resolved in the fragment screen and with new functionally relevant residues. Residue interactions measured from the crystallographic fragment screen (fragments), and predicted interactions for the scaffold inspiration fragments (inspirations), scaffolds, and elaborations are shown. The frequency of interactions of all compounds are shown in the bar chart on top. Residues are grouped by functionally relevant residues,^85,86^ residues where interactions were only found with elaborations, and residues where interactions were found with both fragments and elaborations. GLY 32 (green) is the only functionally relevant residue that was not engaged by fragment screen hits but is predicted to interact with Syndirella elaborations. Circle size represents the frequency of predicted or experimentally observed interactions with each residue. Circle color corresponds to the interaction type where blue is electrostatic, purple for pi-cation, orange for pi-stacking, red for hydrogen bonding, and green for hydrophobic.

Nearly all functionally relevant residues were predicted to result in interactions in the elaboration set, but a new hydrogen bonding interaction with GLY 32 is only found in the elaborations and not in the experimental fragment screen (Fig. 4). These seven functionally relevant residues are grouped in Fig. 4 and were identified by mutational studies (ASP 10, ASN 24, GLY 32, VAL 33, GLY 112, TYR 114, ARG 144).^85,86^ Specifically the single-point mutations G32E and G112E reduced ADP-ribose binding to ∼4% meaning these residues could be an important target for inhibitor development.^85^ Importantly, interactions with GLY 32 were only found with the elaborations and none with the fragments or scaffold originally. This evidence demonstrates that Syndirella's elaboration strategy can uncover interactions with functionally relevant residues that remain hidden in conventional fragment-to-lead optimization approaches, potentially revealing novel inhibitor design opportunities that would otherwise be overlooked through traditional fragment merging alone.

The functional significance of newly explored residues is exemplified in the series shown in Fig. 2, where Syndirella elaborations are predicted to engage mechanistically vital residues through novel interactions. While the experimentally validated interaction with ARG 144—a functionally relevant residue^85^—is maintained from the original fragment c0935a through the scaffold and elaborations, the addition of a hydroxyl group in elaboration 3778 enables a predicted interaction with VAL 33, another key residue^85^ that was not engaged by the inspiration fragments or scaffold.

This comprehensive exploration of interaction space demonstrates Syndirella's effectiveness in generating chemical diversity that is predicted to recapitulate interactions measured in the initial fragment screen and with residues beyond the initial fragment screen, potentially identifying novel SAR with functionally relevant residues.

### Common reactions drive most syntheses, with synthesis coverage improving through tunable, data-driven additions

Syndirella enables prioritization of key chemistry to add to a HT reaction repertoire, expanding accessible chemical space through data-driven analysis. Analysis across all targets revealed that the largest attrition of the original-743 scaffolds is from the explicit synthesis requirements, where 29% did not have a retrosynthesis route returned by Manifold or all routes returned had reactions outside of Syndirella's current repertoire (Fig. S1a and the 23 in-repertoire reactions at the time of this analysis are available as RXN_SMIRKS_CONSTANTS.json on Zenodo^62^). While any HT synthesis platform can benefit from expanding its reaction scope, identifying which new reactions that would most effectively increase chemical space access is crucial for prioritization.

In a separate experiment, all scaffolds were input into Manifold and the top five retrosynthesis routes were returned as ordered by Manifold. Seen in Fig. 5a, Syndirella's reaction repertoire could synthesize over 60% of the original-743 scaffolds. The attrition due to no route returned by Manifold was ∼15% and a loss of a further ∼24% due to all routes containing reactions outside of Syndirella's repertoire.

**Fig. 5:**
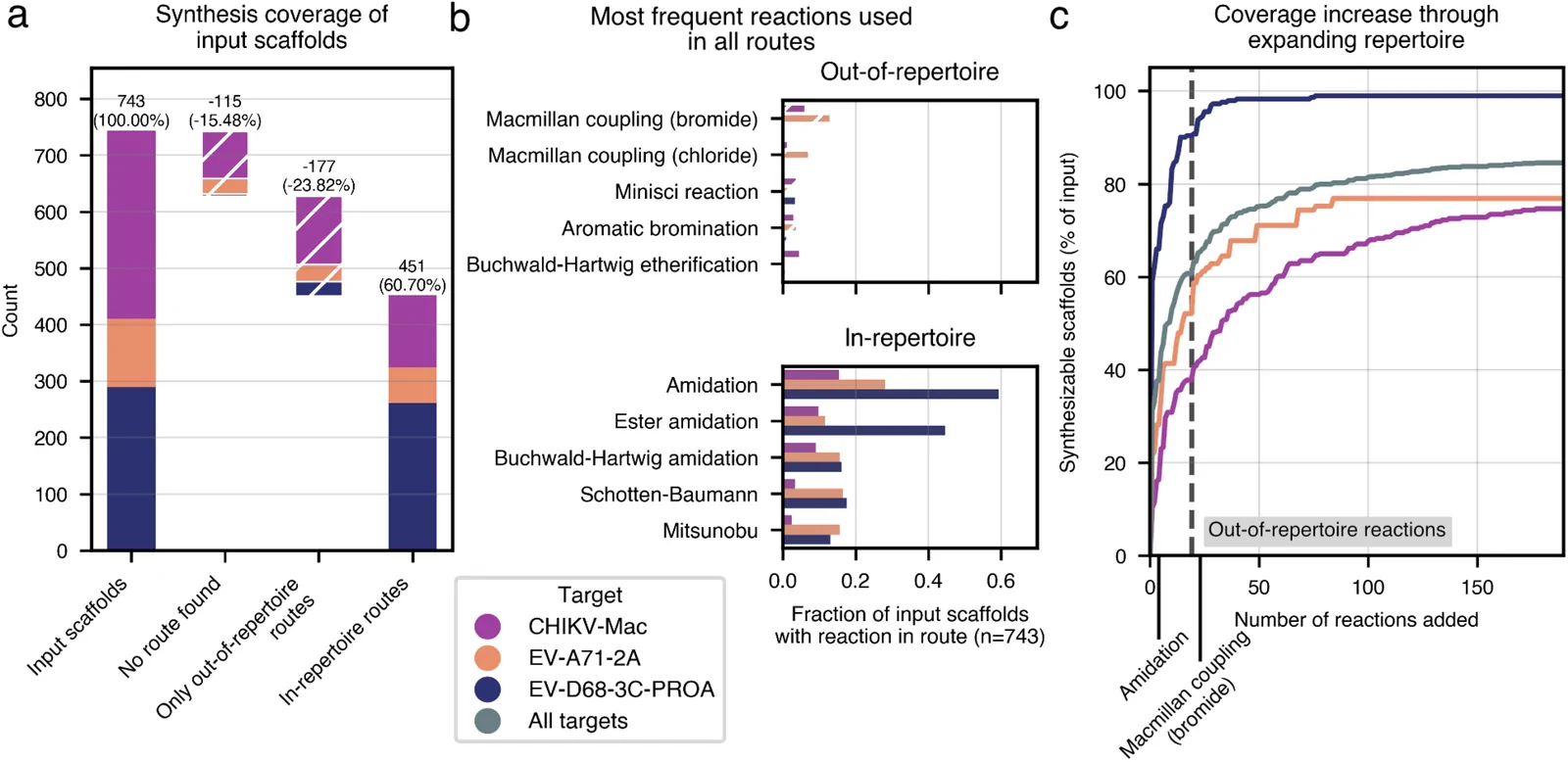
Tractable chemistry can synthesize 60% of input scaffolds, with a clear strategy to increase overall synthesizability. (a) Waterfall of scaffold attrition showing counts (and % of input) lost because Manifold returned no route or only out-of-repertoire routes, leaving scaffolds with ≥1 fully in-repertoire route. (b) Fraction of input scaffolds (*n* = 743) whose routes include a given reaction at least once. Top five reactions are shown separately for out-of-repertoire and in-repertoire groups. (c) Cumulative percent of scaffolds made synthesizable as reactions are added to an empty repertoire. In-repertoire reactions are added first (by decreasing frequency), then remaining out-of-repertoire reactions (after the dashed line).

The majority of scaffolds use reactions already encoded within Syndirella's repertoire. As shown in Fig. 5b, amidation and ester amidation dominate synthesis routes for most targets, with amidation utilized in nearly 60% of EV-D68-3C-PROA scaffolds. In contrast, out-of-repertoire reactions (those not currently encoded in Syndirella at the time of analysis) appear far less frequently, with each used in under 20% of scaffold routes (Fig. 5b). Even so, these reactions still contribute to increasing overall synthesizability when added to the repertoire.

Incorporating high-frequency in-repertoire reactions rapidly recovers the majority of synthesizable scaffolds, whereas adding out-of-repertoire reactions yields a continued but slower rise in coverage, seen in Fig. 5c. This disparity reinforces that simple, robust transformations, such as amidation and ester amidation, dominate the synthesis landscape because they reflect long-standing medicinal chemistry practice^45,87,88^ and perhaps make up a significant portion of Manifold's training data.^89,90^ Additionally, the fragment libraries used to design these scaffolds could be biased towards more sociable fragments (easily synthetically expanded) therefore resulting in these fragment-derived scaffolds to be synthesized by common reactions.^91–93^

CHIKV-Mac scaffolds show a more modest improvement of synthesis coverage overall, likely due to their greater diversity. This diversity stems from an order of magnitude more fragment combination patterns than the other two targets, requiring a broader range of synthetic transformations (Table 1). Among scaffolds with only out-of-repertoire routes, CHIKV-Mac required 132 unique reactions compared to just 39 for the other two targets. Syndirella can query and structure this data required for reaction prioritization using the -just_retro flag, enabling a data-driven approach to identifying which new reactions would most effectively expand synthetic accessibility across multiple targets.

Syndirella also allows synthetic flexibility by enabling users to specify alternative reactions or preferred reactants based on experimental constraints or preferences. This feature directly edits the original reactants in the reaction to be replaced, with built-in checks to only modify reaction atoms in the original reactant. For instance, users can substitute boronic acid reactants with their typically less expensive boronic ester derivatives for Suzuki coupling reactions. The practical impact of the alternative reaction flexibility is substantial, by incorporating amidation as an alternative route consistently yielded more unique products than Schotten–Baumann reactions alone (Fig. S4). For one scaffold, amidation generated 2312 unique products compared to just 153 *via* Schotten–Baumann chemistry, a 1410% increase. This significant expansion in accessible chemical space highlights how Syndirella's tunable reaction preferences serve dual purposes: optimizing synthetic strategy based on practical considerations while potentially accessing a broader range of elaborations.

### Syndirella proposes compounds distinct from traditional superstructure search methods

We compared Syndirella's outputs to traditional superstructure search methods provided by Postera's Manifold (https://postera.ai/) and Arthor (https://arthor.docking.org/) which are both catalog search methods.^94^ Overall, Manifold resulted in the most compounds; >1 M for 211 scaffolds compared to Syndirella which returned <0.5 M for 310 scaffolds (Table S1). Syndirella had the most elaborated scaffolds for CHIKV-Mac and EV-A71-2A, while Arthor had the most scaffolds with superstructures overall with 348 (Table S1). But, Syndirella has the highest breadth-to-depth ratio (7.4 × 10^−4^*versus* 5.1 × 10^−4^ for Arthor and 1.8 × 10^−4^ for Manifold) seen visually in Fig. S5.

Since Syndirella imposes a hard 10 K limit on elaborations (ordered by number of heavy atoms) as a default operating constraint, the same restriction was applied to Arthor and Manifold, removing the largest compounds from all methods equally, resulting in a drastic change to the number of elaborations returned. The Venn diagram of the overlapping elaborations found per method is shown in Fig. 6a with no restriction, showing clear domination by Manifold. Then in Fig. 6b Syndirella clearly produces the most elaborations since Arthor and Manifold decreased by 46% and 84% respectively.

**Fig. 6:**
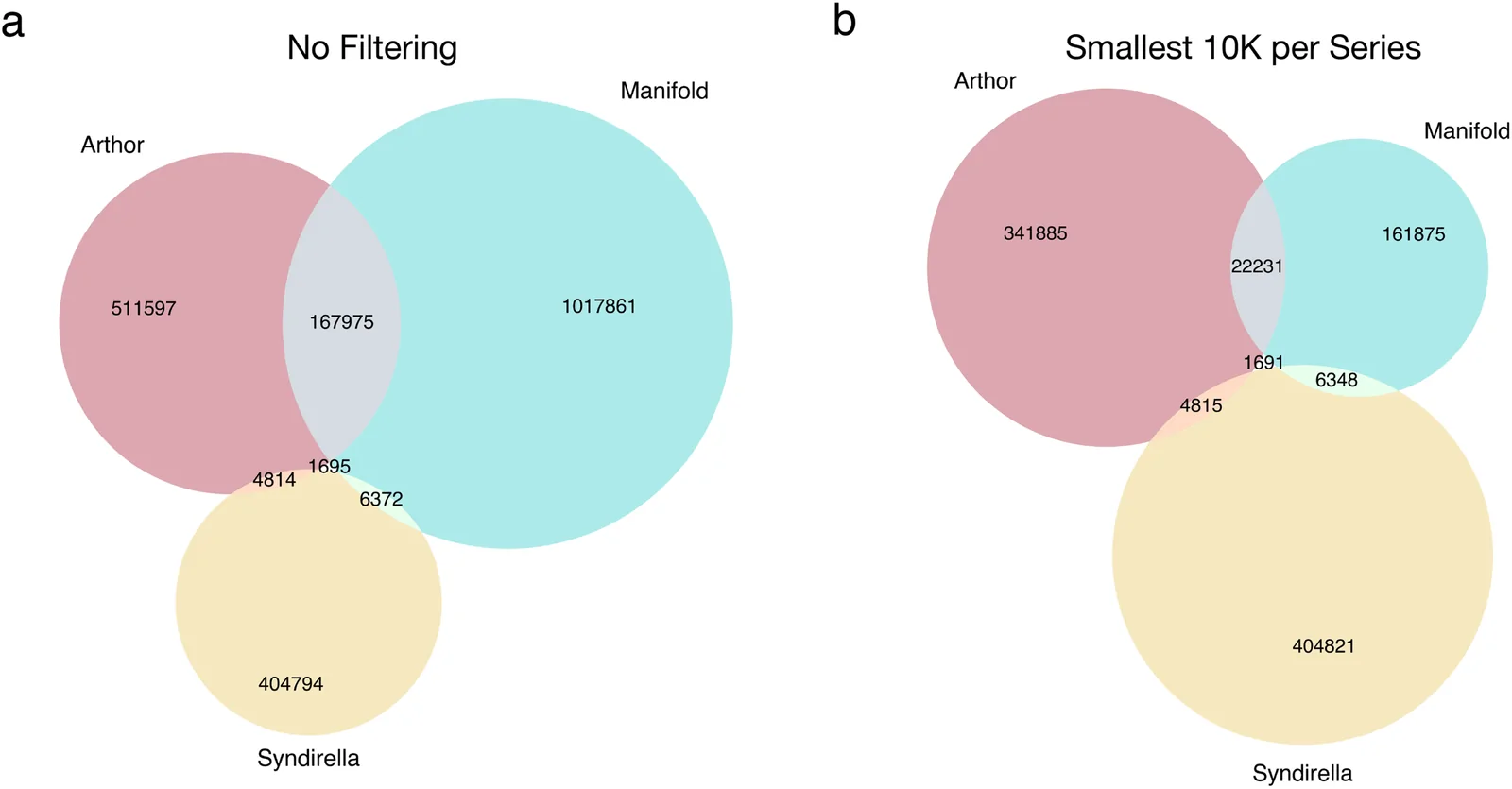
Syndirella covers a distinct chemical space to Arthor and Manifold. (a) Venn diagram of the compounds found labeled by search method for all targets and compared by InChI key. (b) Venn diagram of the top 10 K smallest compounds per series by molecular weight.

Syndirella covered a distinct chemical space to Arthor and Manifold, seen in Fig. 6. While Arthor and Manifold resulted in nearly 168 K compounds combined, Syndirella's overlap with them was less than 13 K. Interestingly, despite the small intersection, the physicochemical property distributions remained similar across all three methods (Fig. S9), suggesting equivalent coverage of property space despite the exploration of different regions of chemical space.

An example series is compared between Arthor and Syndirella in Fig. 7 demonstrating Syndirella outperforms Arthor and Manifold (as Manifold output no superstructures) in generating R-group diversity from the same scaffold. Syndirella produced 1197 unique compounds resulting in 14 pharmacophores whereas Arthor resulted in only 32 compounds with five pharmacophores (Fig. 7a and b).

**Fig. 7:**
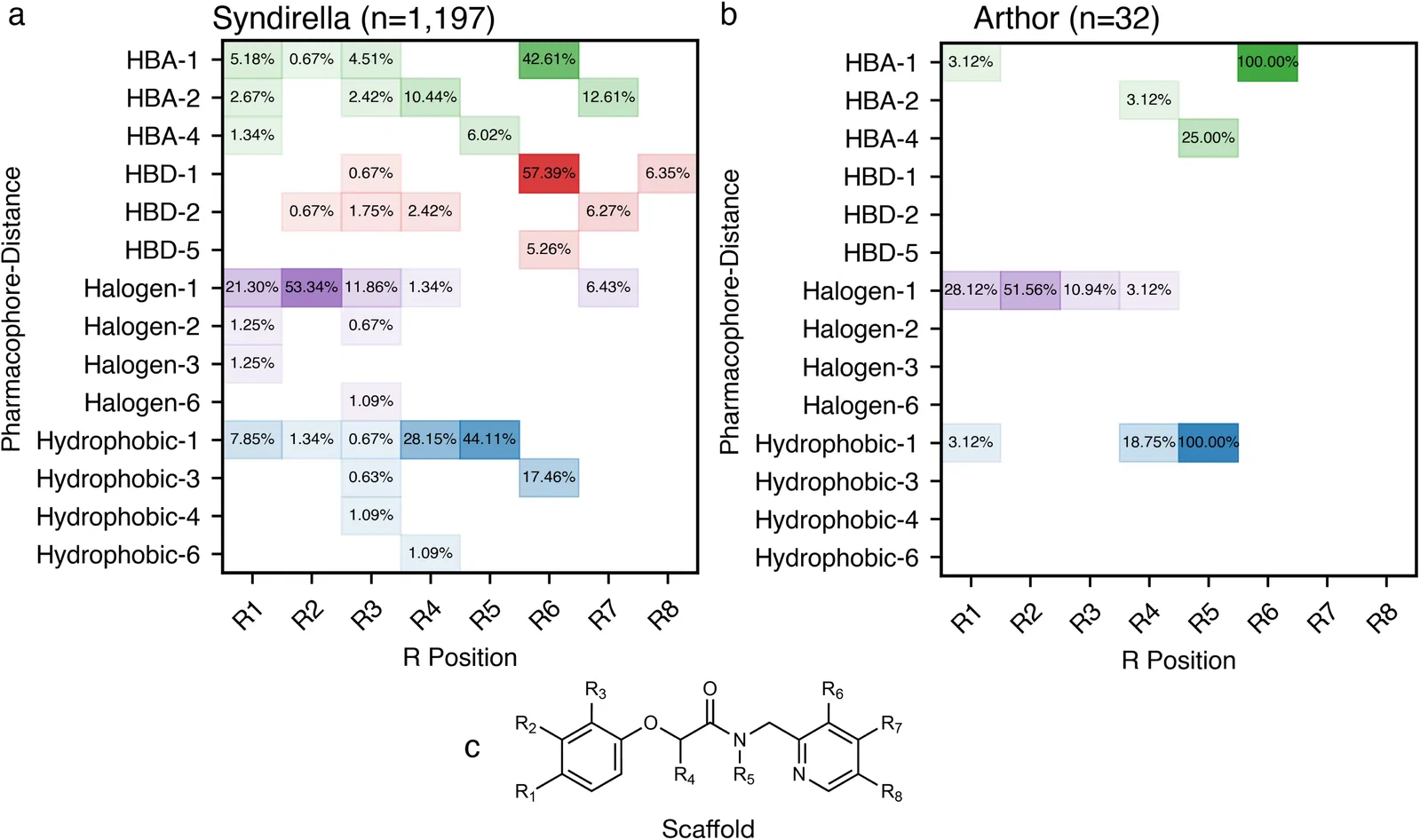
Syndirella can find a larger series and more unique pharmacophores than a superstructure search by Arthor and Manifold. (a) Pharmacophore-distance heatmap for Syndirella generated compounds (*n* = 1197). (b) Pharmacophore-distance heatmap for compounds output by Arthor (*n* = 32). (c) The input scaffold (O=C(N(C)CC1=CC=CC(O)=N1)COC2=CC=CC(F)=C2). No superstructures were returned by Manifold.

### Syndirella produces elaborations that escape the catalog and with generally higher predicted interaction productivity for a given budget than purchasing from the catalog

More products are accessible by purchasing reactants for synthesis compared to purchasing the products directly. Since each elaboration produced by Syndirella has an explicit synthesis route, reactants can be selected for purchase instead of products. From 15 K randomly sampled passing elaborations and scaffolds across all targets (ΔΔ*G* < 0, RMSD < 2 Å to the inspiration fragments, passed intra-geometry PoseBusters checks) we obtained quotes from Enamine (see Methods) to determine the availability of all required reactants, the availability of reactants where each cost less than 100 EUR, and the direct availability of the final product molecule with the results provided in Table 2. The results clearly indicate that synthesizing compounds from purchasable reactants offers a larger set of molecules compared to purchasing the final products directly, as 28.2% of compounds have all reactants purchasable compared to 8.5% of products. This increases the number of accessible compounds by 230% overall.

**Table 2:** Compound purchasability analysis within Enamine

	Passed	Sampled (% of passed)	All reactants purchasable (% of sampled)	All reactants purchasable (reactant price < 100 EUR) (% of sampled)	Product purchasable (% of sampled)
CHIKV-Mac	78 063	5000 (6.4%)	**1524** (30.5%)	768 (15.4%)	279 (5.6%)
EV-A71-2A	86 416	5000 (5.8%)	**1090** (21.8%)	816 (16.3%)	336 (6.7%)
EV-D68-3C-PROA	16 406	5000 (30.5%)	**1617** (32.3%)	579 (11.6%)	666 (13.3%)
Total	180 885	15 000 (8.3%)	**4231** (28.2%)	2163 (14.4%)	1282 (8.5%)

Purchasing reactants provides access to nearly equivalent chemical space as direct product procurement, while simultaneously expanding accessible regions. The t-SNE visualization in Fig. 8a demonstrates substantial spatial overlap between products accessible *via* reactant purchase (gold) and directly purchasable products (green), with the reactant strategy offering access to previously inaccessible chemical space regions. For example, similar compounds accessible by both methods are circled on the right and a cluster of compounds only accessible by reactant purchasing are circled in the middle. The overlap between procurement strategies is quantified in the Venn diagrams (Fig. 8b and c), which show significant intersection between the two procurement strategies, with over 90% of directly purchasable products also accessible through reactant purchase (see Fig. S6 for by target availability). These findings suggest that reactant-based procurement represents a viable alternative strategy that maintains coverage of established chemical space while expanding synthetic accessibility to novel regions.

**Fig. 8:**
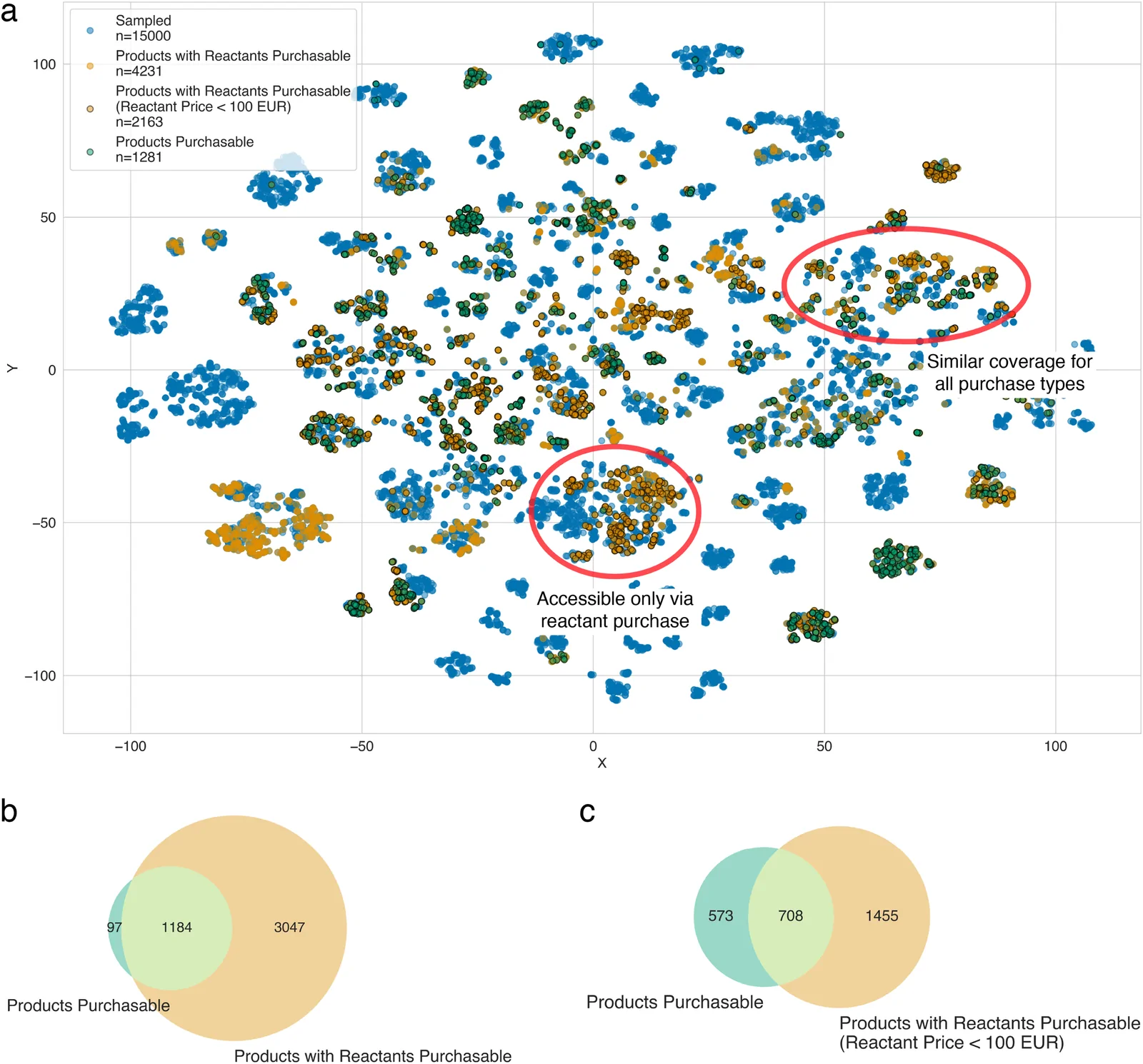
Reactant purchasing expands accessible chemical space beyond direct product procurement. (a) The chemical space of the 15 K sampled compounds projected onto two dimensions using the t-SNE algorithm and colored by availability status in Enamine after quoting. (b) Venn diagram of the compounds accessible through Enamine through either the products purchasable or all reactants purchasable route. (c) Venn diagram of accessible compounds with all reactants restricted to a price of <100 EUR.

We next asked whether the increased accessibility of compounds *via* reactant procurement is maintained under realistic budget constraints. For a given budget, more compounds are accessible by purchasing reactants and synthesis in-house than by purchasing final products, even after accounting for experimental synthesis costs and synthesis failure. The raw price of all reactants required to synthesize products (reactant price < 100 EUR) *versus* price of the product directly is 62.6% cheaper (Fig. S7). Including an estimated experimental synthesis cost of 6.11 EUR per compound for the reactant procurement approach (Table S4), the reactant-based strategy still enables access to more compounds. Including the experimental synthesis cost and when all reactants are under 100 EUR and given a budget of 10 K EUR, 642 compounds can be accessed by purchasing reactants compared to 144 by purchasing directly (Fig. S8a). Additionally, accounting for synthesis failure using the 55.5% success rate from the NCS-1 application^50^ results in 356 successfully synthesized compounds from the 642 attempted. While this is a context specific synthesis success rate, it suggests that realistically, reactant procurement can access nearly 2.5 times more compounds compared to 144 available by direct purchase.

Beyond raw compound count, reactant procurement can result in broader coverage within scaffold series and shorter overall lead times. Reactant purchasing yields 11.5 compounds per scaffold series on average *versus* 4.8 for product purchasing. Lead times are also shorter where in the overlapping set, 100% of products have reactants deliverable within 2 to 3 weeks, compared to fewer than 5% of the directly purchasable products (Table S2).

Additionally, purchasing reactants can potentially yield higher interaction productivity than purchasing products directly, even when experimental synthesis costs are included. We define predicted interaction productivity as the number of unique predicted protein–ligand interactions achievable at a given budget. As shown in Fig. 9, we plot, for each target, the number of unique predicted interactions *versus* budget for both procurement methods. For EV-A71-2A and EV-D68-3C-PROA, the reactant-based strategy has higher predicted interaction productivity than direct product purchasing at every budget. At a 10 K EUR budget for EV-A71-2A, 268 products synthesized from purchased reactants yield 49 predicted interactions, compared to 138 directly purchased compounds with 26 predicted interactions (Table S5). For CHIKV-Mac at a 10 K EUR budget, however, the product purchased set yields more predicted interactions than the reactant purchased set (42 *vs.* 38) with fewer compounds (119 *vs.* 276). This suggests that some product purchasable sets may already be relatively efficient in terms of interaction productivity. Although the interaction productivity increases at a higher rate for the reactant purchasing strategy than the product purchasing after spending 10 K EUR. For CHIKV-Mac, spending an additional 40 K EUR results in only 2 additional predicted interactions *via* the product purchasing strategy *versus* 17 for the reactant purchasing strategy. This example highlights how users can make informed decisions about budget increases and understand how much additional value they may gain.

**Fig. 9:**
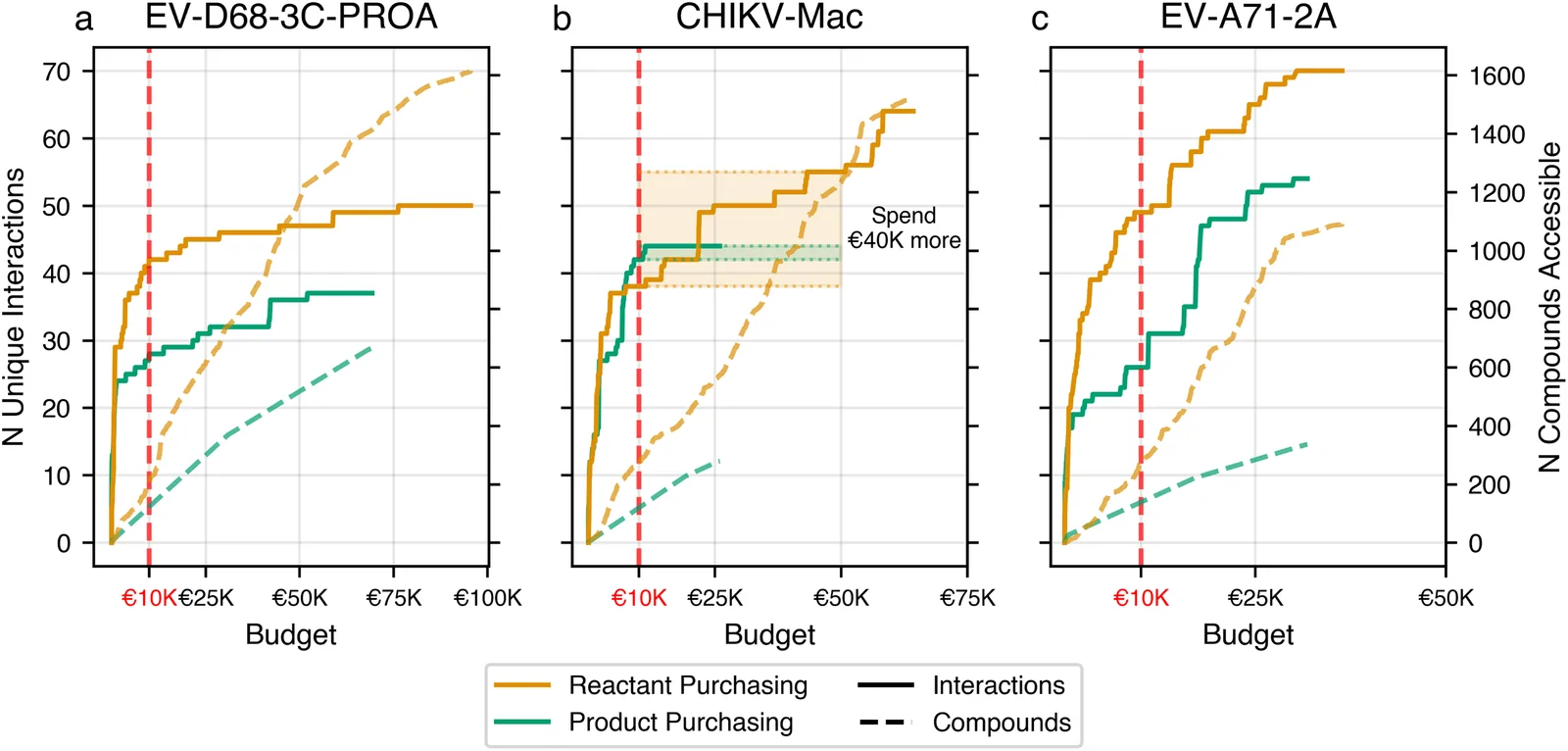
Purchasing reactants for synthesis generally yields higher predicted interaction productivity than purchasing final products, even when experimental synthesis costs are included. We define predicted interaction productivity as the number of unique predicted protein–ligand interactions achievable at a given budget. For each target, compounds are ordered by increasing total cost of the lowest-cost acquisition route (final product price for product purchasing, or sum of reactant prices plus a per-compound synthesis cost of 6.11 EUR for reactant purchasing, see Table S4). Curves show, as a function of budget, the cumulative number of unique predicted protein–ligand interactions (solid lines, left *y*-axis) and compounds (dashed lines, right *y*-axis) that can be accessed by product purchasing (green) or reactant purchasing (gold). Panels (a–c) correspond to EV-D68-3C-PROA, CHIKV-Mac, and EV-A71-2A targets, respectively. In all panels, the vertical red dashed line marks a budget of 10 K EUR. (b) the shaded regions show the increase in number of interactions found in product sets from a 10 K to 50 K EUR budget.

More generally, predicted interaction productivity depends strongly on how reactants (and hence products) are selected under a fixed budget. Methods such as HIPPO (used in this work, see https://github.com/mwinokan/HIPPO) and SPARROW^44^ explicitly integrate reactant cost, expected synthetic cost, and product-level rewards like predicted interaction productivity to jointly optimize which molecules to synthesize. While a full comparison of such advanced selection strategies is beyond the scope of this manuscript, under our simple price-ordered selection scheme the reactant-based strategy typically yields higher predicted interaction productivity than direct product purchasing.

Overall, these findings demonstrate that Syndirella's reactant-based purchasing strategy not only accesses a broader, catalog-escaping chemical space than direct product purchasing, but also can deliver higher predicted interaction productivity.

### Experimental validation of Syndirella results in SAR elucidation using structural and kinetic measurements

Syndirella was applied to elaborate fragment derivatives for the calcium binding protein NCS-1 to explore the binding region and the SAR of novel fragment inspired chemical matter. As detailed in the companion paper, Muñoz-Reyes *et al.*,^50^ twelve scaffolds were elaborated using Syndirella, producing thousands of designs which were downselected based on predicted interaction diversity, reactant price, and other metrics. From the explicit synthesis route definition by Syndirella, this allowed for reactant purchasing for in-house array synthesis and finally direct-to-biology testing of the crude reaction mixtures by X-ray crystallography and kinetic measurement by grating-coupled interferometry (GCI). Full synthetic procedures, product characterization, and purity analysis for these compounds are reported in the companion paper.^50^ This pipeline, seen in Fig. 10a, can be applied to any structure-based drug discovery project with fragment information.

**Fig. 10:**
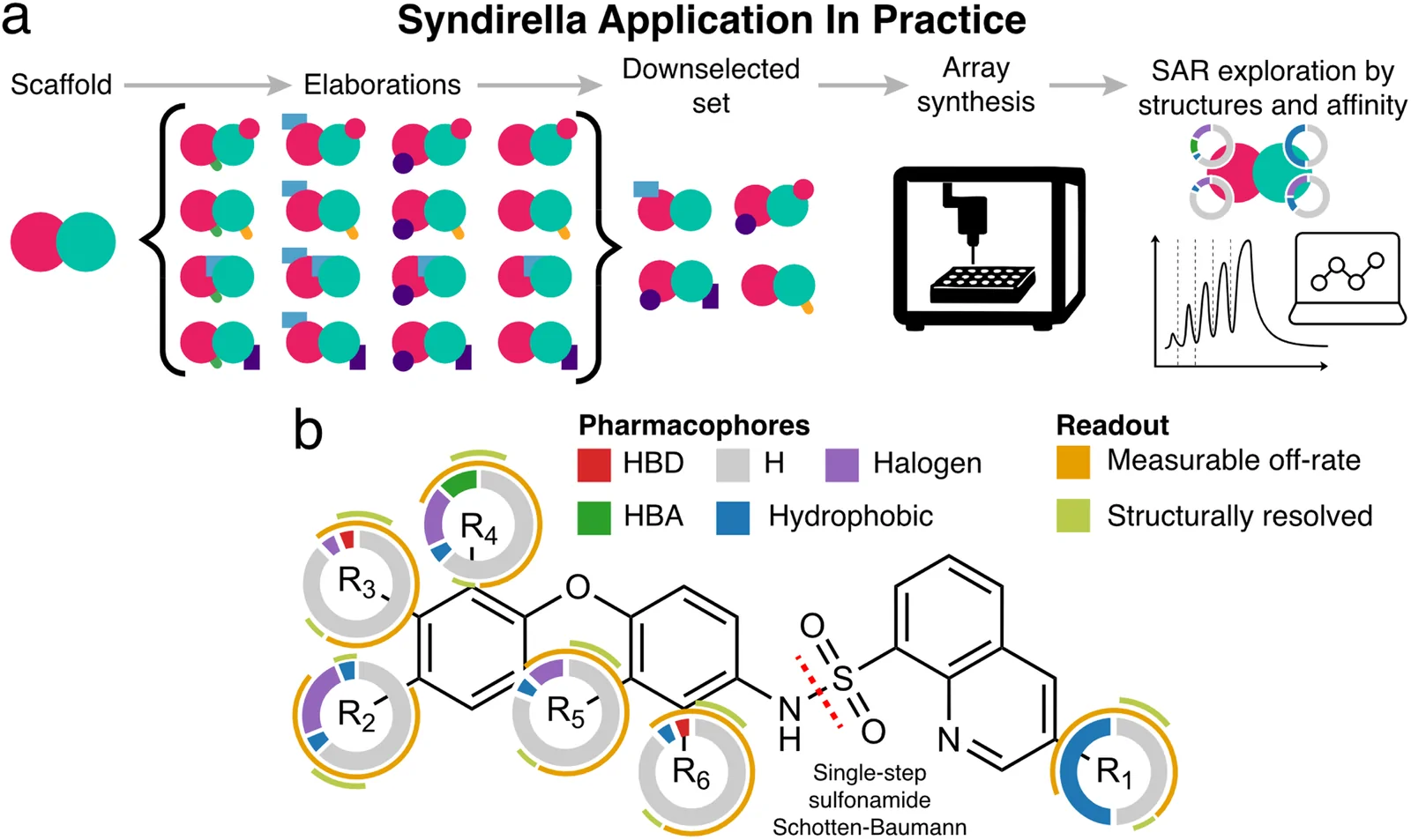
Syndirella is used to provide SAR exploration for real-world drug development. (a) A schematic layout of Syndirella usage in practice, starting from a scaffold compound, generation of elaborations, downselection using any method, array synthesis using the explicit building blocks, and finally structural and kinetic measurements allowing for SAR elucidation. (b) An example SAR analysis for one scaffold from the NCS-1 application.^50^ The scaffold was elaborated with sulfonamide Schotten–Baumann chemistry and resulted in a series of 16 compounds with six variable R positions. Doughnut plots are shown for each R position displaying the fraction of compounds with the labeled pharmacophore type, grey for a single hydrogen, blue for a hydrophobic group, purple for a halogen, red for a hydrogen bond acceptor (HBA), and green for a hydrogen bond donor (HBD). The outer rings encode experimental readouts aggregated over variants at that position: orange indicates the fraction of compounds with measurable off-rates by GCI and light green indicates the fraction for which a structure was resolved by X-ray crystallography. Overlapping rings means those compounds had readouts by both methods.

To highlight how Syndirella advanced the SAR elucidation for the work in Muñoz-Reyes *et al.*,^50^ we here show an in-depth R-group analysis for one of the scaffolds (Ric8enhan-3) which is summarized in Fig. 10b. Across the 16-member series, five R-group pharmacophore classes were sampled (H, halogen, hydrogen-bond donor, hydrogen-bond acceptor, and hydrophobic). Readouts were summarized as “measurable off-rate” for kinetics and “structurally resolved” for crystallography where the outer rings on the doughnut plots show the pharmacophores resulting in those readouts. Here, measurable off-rate means that GCI returned an off-rate estimate and structurally resolved means a crystal structure of the ligand was resolved by X-ray crystallography, see Methods of the companion paper Muñoz-Reyes *et al.*^50^ for details.

Using these definitions in this work we found, 2/16 compounds (∼13%) showed both measurable off-rates and a resolved structure, 1/16 (∼6%) produced a structure without a measurable off-rate, 9/16 (∼56%) showed measurable off-rates without a structure, and 4/16 (∼25%) showed neither. The partial overlap and frequent separation between kinetic and structural success yields a useful orthogonal dataset for future methodological SAR analysis (beyond the scope here; see, *e.g.*, recent SAR-elucidation frameworks^34^). At a high level, substitutions that introduced hydrophobic features at R_1_ did not yield a crystal structure compared to those which contained a single hydrogen. By contrast, varying R_3_ and R_4_ toward donor/acceptor character was more often associated with productive outcomes (instances with both structural and kinetic evidence were observed among these classes). These qualitative rules, derived from a pharmacophore-diverse elaboration set, can guide subsequent design.

Rather report the median rather than the mean since that was the value reported in the companion paper.^50^ The median RMSD between predicted and observed ligand poses was 4.60 Å, and 7 of 16 experimental poses shared at least one protein–ligand interaction with the corresponding prediction.

The reactant-based purchasing strategy enabled by Syndirella resulted in significant improvements in chemical space exploration while reducing costs compared to direct product procurement. To perform the analyses for this manuscript, we queried the 5335 elaborations in MCule^59^ and found only two compounds could be purchased for 295.86 EUR (average of 147.93 EUR per compound), demonstrating the limited availability of elaborations through traditional suppliers. In contrast, the reactant-based approach allowed for the potential synthesis of 454 compounds from purchasing 212 reactants for 7557.39 EUR. Including synthesis labor and materials cost of 3053.00 EUR to synthesize (Table S4), the cost totals to 10 610.39 EUR (23.37 EUR per compound). Of the 454 compounds attempted, 252 were successfully synthesized, resulting in a final price of 42.10 EUR per compound—71% lower per compound than direct purchasing while providing access to two orders of magnitude more compounds that would otherwise be commercially unavailable.

## Discussion

We have demonstrated that Syndirella systematically generates pharmacophorically diverse congeneric series that enable comprehensive SAR exploration from crystallographic fragment screens. Through analysis of multiple targets (CHIKV-Mac, EV-A71-2A, EV-D68-3C-PROA) and experimental validation with NCS-1,^50^ our method produces series that are predicted to sample new binding site interactions relative to inspiration fragments. As exemplified with CHIKV-Mac, elaborations resulted in a predicted interaction with the functionally important residue GLY 32, not measured in the fragment screen (Fig. 4). While providing less pharmacophore coverage than real-world heterogeneous approaches like in the COVID Moonshot (Fig. 3), this systematic enumeration offers a tractable method for SAR studies supported by HT chemistry.

The NCS-1 experimental case study (detailed in Muñoz-Reyes *et al.*^50^) and the more detailed SAR analysis shown here, demonstrated how Syndirella was used for preliminary SAR elucidation. For one scaffold, both favorable pharmacophores (hydrogen bond acceptors/donors at R_3_ and R_4_) and unfavorable modifications (hydrophobic groups at R_1_ preventing crystallization) were identified. This experimental validation shows how Syndirella is used to help understand SAR early in the drug design process, such as identifying activity cliffs and position-specific functional group effects.^95,96^

However, the brute-force combinatorial approach of reactants has inherent limitations that we address through conservative risk management strategies. The static 10 K elaboration threshold used in this work creates tradeoffs where scaffolds accommodate either few R-positions (<4) with many unique groups (>100) or *vice versa* (Fig. S10). To manage synthetic and design risk—which we define as positively correlating with atoms added relative to scaffolds, encompassing uncertainty about whether elaborations will be successfully synthesized, adopt inspiration fragment conformations, or exhibit measurable biological effects^51,97^—we prioritize compounds with fewer novel atoms. Since scaffolds already differ from constituent fragments through merging, this conservative threshold maintains lower risk while necessarily excluding larger functional groups. Future iterations could implement position-specific elaboration or variable thresholds.

A key strength of Syndirella lies in leveraging established medicinal chemistry reactions while providing unprecedented user flexibility for HT workflows. Analysis reveals 74% of successful routes incorporate at least one of the top five medicinal chemistry reactions identified by Brown and Boström:^87^ amide formation, Suzuki–Miyaura coupling, S_N_Ar, Boc protection/deprotection, and electrophile–amine reactions. While Syndirella uses SMIRKS to represent reactions, these patterns only capture connectivity and cannot encapsulate the complexities of real-world synthesis such as solubility, by-product formation, chemoselectivity, and regioselectivity. This is a limitation of template-based transformations generally,^98^ and predicting these outcomes *in silico* remains an open and active area.^99^

Syndirella nonetheless offers flexibility absent from existing enumeration tools,^40,41,100^ allowing users to encode specific chemistry workflows, modify retrosynthetic routes, and select cost-effective reactants. A natural extension of this flexibility is to inform it with experimental synthesis outcomes. These outcomes could be analyzed to identify reactant features associated with synthetic success or failure. Such features could be encoded as route or reactant filters within Syndirella's existing preference framework, allowing the synthetic experience captured in one campaign to constrain and prioritize the routes proposed for the next. Curated SMARTS libraries such as SMARTS-RX^64^ already support this, flagging building blocks that match more than one reactive pattern, such as an amine intended for amide coupling that also carries a second amine or a carboxylic acid, and so risk poor regioselectivity or side products.

When compared to catalog-based approaches under similar constraints, Syndirella demonstrates superior breadth-to-depth ratio of scaffold congeneric series (Fig. S5). Despite generating fewer total elaborations due to the 10 K limit in this work, Syndirella produces 26.5% more products per scaffold than Arthor and 47.9% more than Manifold under similar restrictions (Table S1). This superior depth-to-breadth ratio (Fig. S5) proves crucial for early development, where comprehensive scaffold sampling prevents premature limitation to low-performing series.

The practical advantages of reactant-based synthesis are not limited to cost. With a 230% increase in accessible compounds through reactant purchasing *versus* direct procurement (Table 2), Syndirella enables access to chemical space around scaffolds that is absent from commercial catalogs, consistent with the observations of Luttens *et al.*^16^ At a 10 K EUR budget and including the realistic constraints of in-house synthesis cost and synthesis failure, this strategy provides access to 356 compounds compared to 144 through direct purchase (Fig. S8), and these estimates are in line with NCS-1 costs of 42.10 EUR per compound for synthesis *versus* 147.93 EUR per compound from MCule. Reactant-based purchasing also lessens a campaign's dependency on supplier capacity for synthesis as only building-block availability is required. Changes in a supplier's capacity, pricing, or lead times can therefore be accommodated by sourcing reactants from a different supplier. Synthesis performed in-house additionally accumulates expertise within the group, which outsourcing does not, so subsequent campaigns benefit from chemistry the group has already run.

This approach carries upfront equipment costs that the per-compound figures above do not capture. The synthesis costs presented come from technician, reagent, and consumables costs (Table S4) and do not include the costs of establishing robotic synthesis capabilities, estimated to total 36 600.00 EUR, see Table S3. The largest costs are the liquid-handling robot (here from Opentrons https://opentrons.com/) and the reactor labware for temperature control, contributing 33% and 53% respectively to the total equipment cost. For a laboratory already undertaking synthesis, these are the main additional equipment investments.

Because the upfront equipment cost is amortized across projects, its per-compound contribution falls as more compounds are synthesized. For the overlapping set of 708 products available both by direct purchase and by synthesis from reactants under 100 EUR (Table S2), the reactant route costs 48.12 EUR per compound (reactant price plus 6.11 EUR synthesis cost) *versus* 88.11 EUR per compound to purchase directly, a saving of approximately 40 EUR per compound. At this estimated saving, the upfront equipment cost of 36 600.00 EUR is recovered after synthesizing 915 compounds, the scale of a few synthesis campaigns. The in-house HT synthesis approach also involves working with crude mixtures rather than purified solutions, but such mixtures remain compatible with crystallographic, biochemical, and biophysical screening formats.^31–33,50^

Beyond accessing more compounds, Syndirella has the potential to improve the productivity of the resulting experiments. By enabling a reactant-based purchasing strategy, it can increase predicted interaction productivity, that is, the number of unique predicted protein–ligand interactions achievable within a given budget (Fig. 9). In our analyses, this means that for the same expenditure, more compounds can be tested and those compounds are predicted to result in a larger set of unique protein–ligand interactions. This can provide more data points per screening campaign and may increase the likelihood of identifying higher-affinity binders.

Syndirella generates rich datasets intrinsically suited for multi-objective optimization, mapping extensive residue contacts (Fig. 4) with explicit synthetic routes for cost-weighted optimization.^44^ By providing comprehensive synthetically tractable compound sets, Syndirella enables more thorough SAR exploration than traditional smaller and more expensive commercially-sourced approaches. Beyond FBDD, Syndirella's approach to systematic SAR exploration around any scaffold makes it equally applicable to non-FBDD direct-to-biology campaigns requiring comprehensive congeneric series generation.

## Conclusion

Syndirella addresses a critical gap in FBDD where current reaction based enumeration tools to produce congeneric series of compounds are not designed to leverage the advantages of HT micro- and nano-scale chemistry platforms. Our analysis across three targets (CHIKV-Mac, EV-A71-2A, and EV-D68-3C-PROA) shows that not only can Syndirella produce congeneric series for SAR analysis that allow for higher diversity by purchasing reactants instead of products, it also could provide a rich dataset to sample binding site interactions. Application to NCS-1 shows how Syndirella allows for direct-to-biology testing for SAR exploration of crystal structure and kinetic data overlap.

In conclusion, Syndirella represents a valuable addition to the FBDD toolbox, offering a practical solution for generating diverse, synthetically accessible elaborations while providing rich SAR information through comprehensive exploration of the binding site. Its integration of HT synthesis considerations makes it particularly well-suited for modern drug discovery workflows where speed, cost, and synthetic accessibility are crucial factors.

## Author contributions

K. K. F. developed the Syndirella methodology and software; performed formal analysis and data curation; generated visualizations; and wrote the original draft of the manuscript. M. W. contributed to methodology and software development and reviewed and edited the manuscript. J. A. M. supervised and reviewed and edited the manuscript. C. M. D. and F. v. D. supervised the research and review and editing of the manuscript. W. T. conceptualized the study; contributed to methodology and software development; supervised the research; and reviewed and edited the manuscript.

## Conflicts of interest

C. M. D. discloses membership of the Scientific Advisory Board of Fusion Antibodies plc and AI proteins, and is a founder of Dalton Tx. The remaining authors declared that this work was conducted in the absence of any commercial or financial relationships that could be construed as a potential conflict of interest.

## Supplementary Material

SC-OLF-D6SC02032A-s001

SC-OLF-D6SC02032A-s002

## Data Availability

Syndirella is found at the GitHub repository https://github.com/oxpig/syndirella. The 23 reaction SMIRKS used in this analysis, all input scaffolds, compounds generated, and summary statistics for each of the three targets, CHIKV-Mac, EV-A71-2A, EV-D68-3C-PROA are located at https://doi.org/10.5281/zenodo.21297861. We predict these datasets to be useful for the development of compound sampling methods considering synthesis and pose information. Supplementary information (SI): tables of superstructure search results and enamine availability, pricing and synthesis cost analyses, and figures covering pipeline attrition, series diversity, per-target chemical space coverage, budget-accessibility tradeoffs, and per-target similarity distributions. See DOI: https://doi.org/10.1039/d6sc02032a.
