# Supplementary material for: Syndirella: synthesis-directed fragment elaboration enables extensive and cost-effective binding site exploration beyond catalog compounds
Source: Chem Sci. 2026 Sep 29;17(41):20118–39. doi: 10.1039/d6sc02032a (PMC13656017; doi:10.1039/d6sc02032a)
Supplement: Supplementary file 1 [file SC-017-D6SC02032A-s001.pdf]

Supplementary Information (SI) for Chemical Science.
This journal is © The Royal Society of Chemistry

Supplementary Information for Syndirella: Synthesis-directed fragment elaboration enables extensive binding site exploration beyond catalog compounds

Kate K. Fieseler[1,2,3], **Max Winokan**[2,4], **Joseph A. Morrone**[5], **Charlotte M. Deane**[1], **Frank von Delft**[2,3,4,6], and **Warren Thompson**[2,4,*]

[1]Oxford Protein Informatics Group, Department of Statistics, University of Oxford, Oxford OX1 3LB, UK

[2]Research Complex at Harwell, Harwell Science and Innovation Campus, Didcot OX11 0FA, UK

[3]Centre for Medicines Discovery, University of Oxford, Oxford OX3 7DQ, UK

[4]Diamond Light Source, Harwell Science and Innovation Campus, Didcot OX11 0QX, UK

[5]IBM TJ Watson Research Center, IBM Research, 1101 Kitchawan Rd., NY, 10598, Yorktown Heights, USA

[6]Department of Biochemistry, University of Johannesburg, Johannesburg 2006, ZA

[*]Corresponding author: warren.thompson@diamond.ac.uk

Supplementary Tables

Table S1: Scaffolds and Elaborations Found by Superstructure Search Methods

	Scaffolds			Elaborations			Elaborations (smallest 10K)		
	Syndirella	Arthor	Manifold	Syndirella	Arthor	Manifold	Syndirella	Arthor	Manifold
CHIKV-Mac	**70**	54	51	149827	241982	**314315**	**149827**	129325	97540
EV-A71-2A	**44**	40	25	151729	320879	**863089**	**151729**	119265	77562
EV-D68-3C-PROA	196	**254**	135	116119	**123368**	17117	116119	**122041**	17117
Total	310	**348**	211	417675	686229	**1194521**	**417675**	370631	192219

Table S2: Products and Reactants Analysis for the Overlapping Set of Products Available by Enamine

	**No restriction**	**All reactants < 100 EUR**
Number of products	1,184	708
Total reactants used	2,368	1,416
Unique reactants used	1,188	713
Total reactants price (no repeat) EUR (per product)	57,078.9 (48.21)	17,177.7 (24.26)
Total reactants price (with repeat) EUR (per product)	116,849.4 (98.69)	29,744.0 (42.01)
Total products price EUR (per product)	117,263.8 (99.04)	62,384.5 (88.11)
Products with 2-3 weeks lead time	2.70%	4.24%
Reactants with 2-3 weeks lead time	95.30%	100.00%

Table S3: Upfront Equipment Cost Estimation for Opentrons Robotic Synthesis

**Item**	**Cost (EUR)**
Opentrons	12,000.00
Opentrons pipettes single channel	2,200.00
Opentrons pipettes multi channel	2,900.00
Reactor labware	19,500.00
**Total**	**36,600.00**

Note: Pricing data collected July 2026.

Table S4: Cost Estimation for Synthesizing 500 Compounds in 1 Week

Item	Unit Cost (EUR)	Unit	Total Cost (EUR)	Cost per Compound (EUR)
Technician	89,175.00	Year	1,714.00	3.43
Lab utilities	238.00	Day	1,189.00	2.38
Tips	9.00	Tip	90.00	0.18
Solvents	24.00	Liter	12.00	0.02
Labware	12.00	Plate	48.00	0.10
**Total**			**3,053.00**	**6.11**

Table S5: Summary Statistics at a 10K EUR Budget for Each Target

Target	Purchasing strategy	N Compounds	N Unique Interactions	Budget (EUR)
EV-D68-3C-PROA	Product	122	28	9,980.30
EV-D68-3C-PROA	Reactant	203	42	9,987.92
CHIKV-Mac	Product	119	42	9,992.30
CHIKV-Mac	Reactant	276	38	10,003.76
EV-A71-2A	Product	138	26	10,001.10
EV-A71-2A	Reactant	268	49	9,842.31

Supplementary Figures

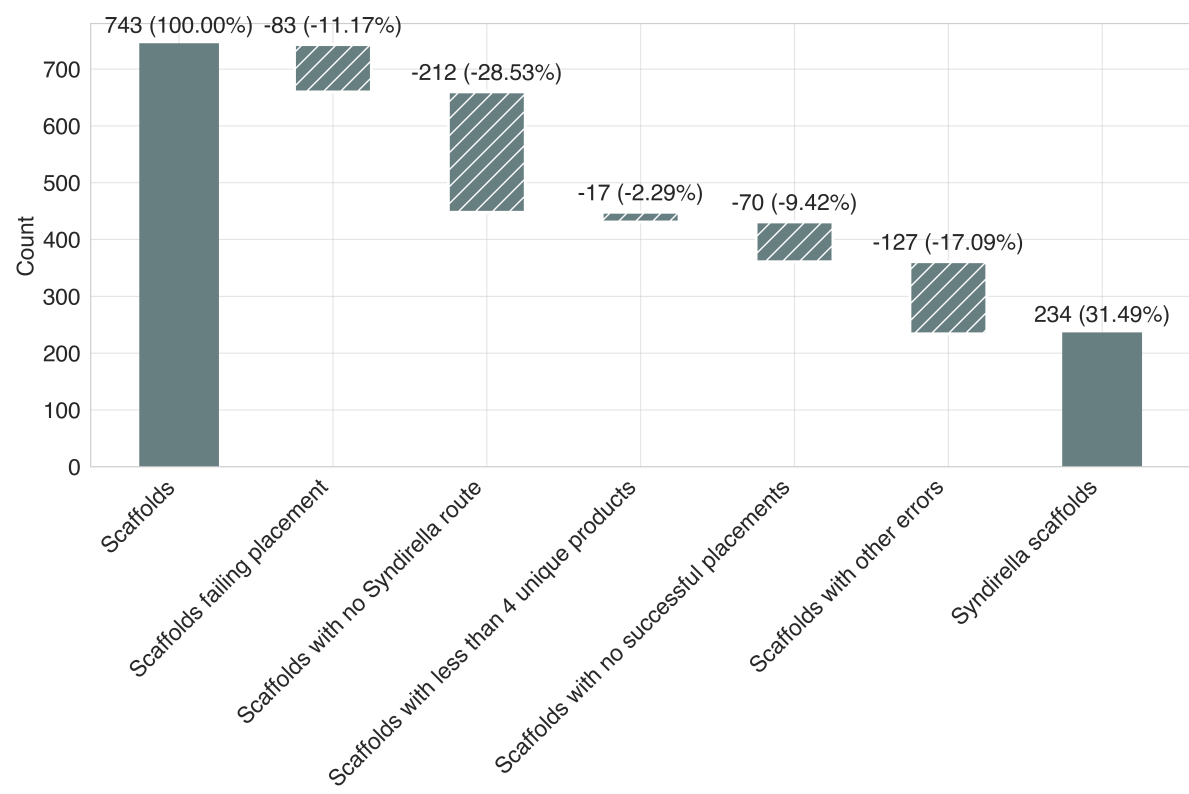

(a)

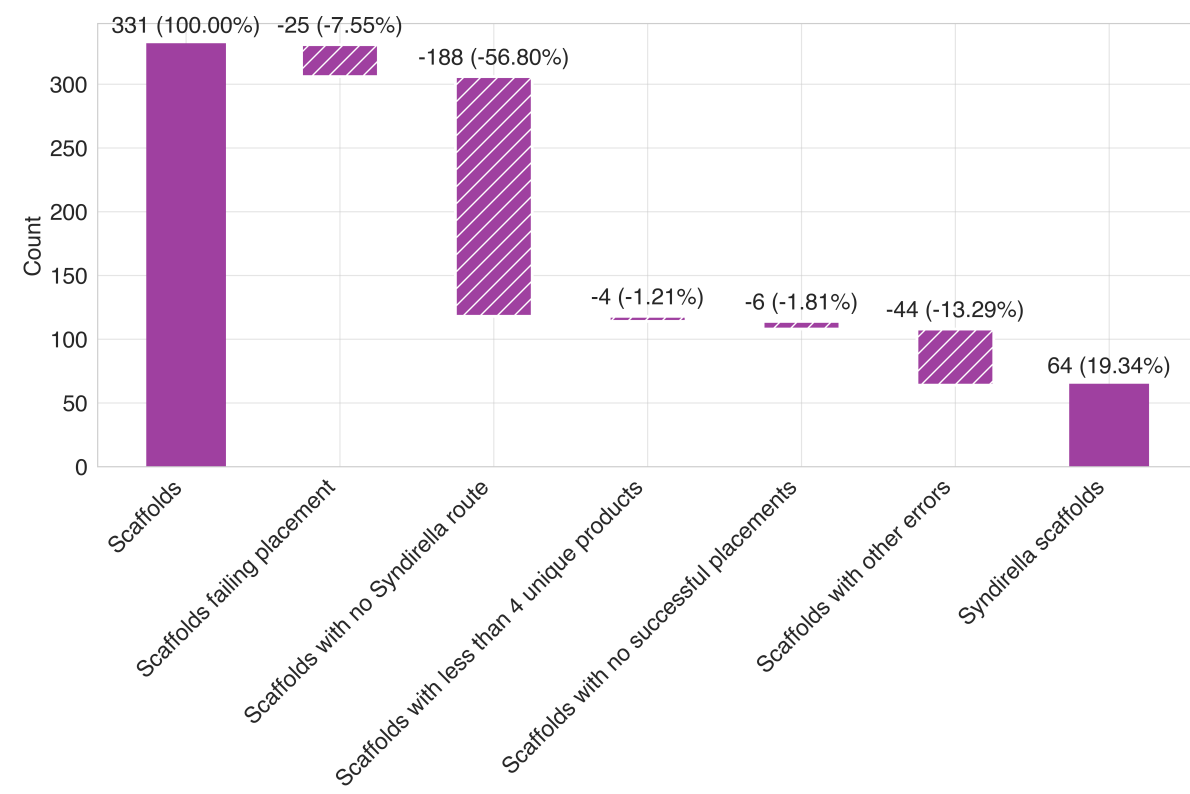

(b)

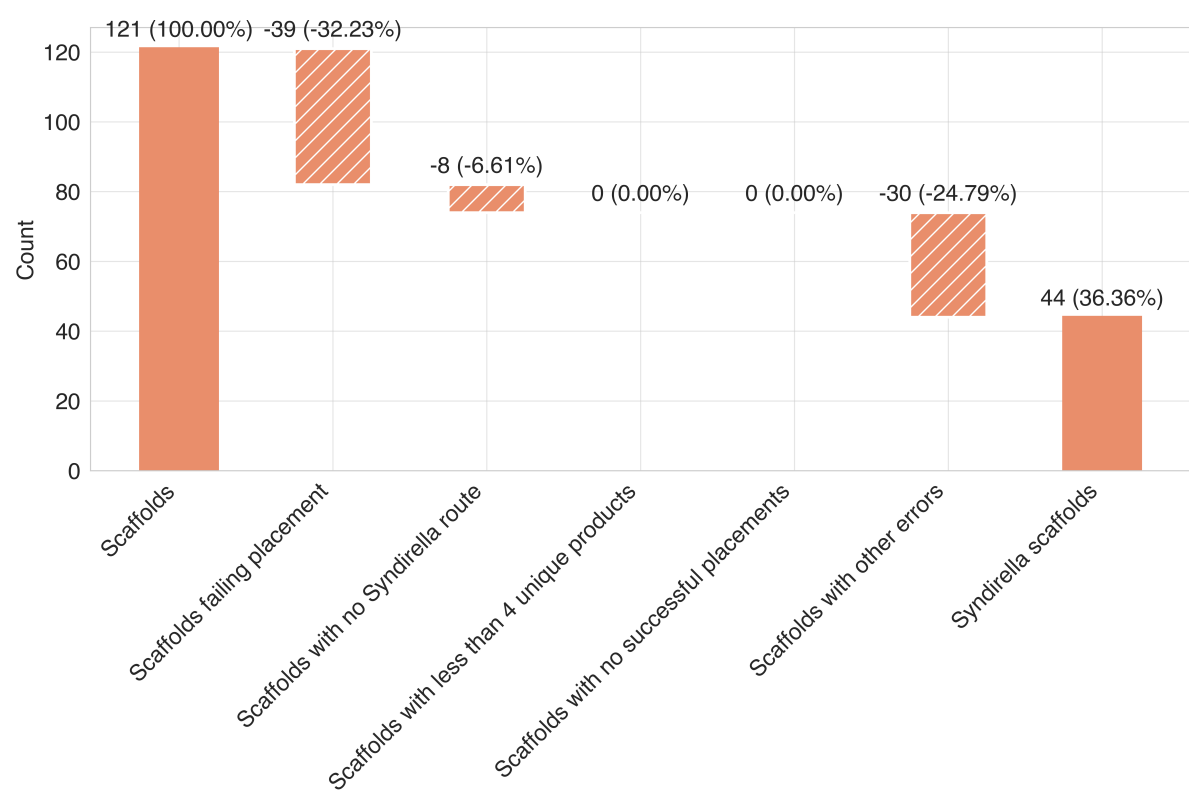

(c)

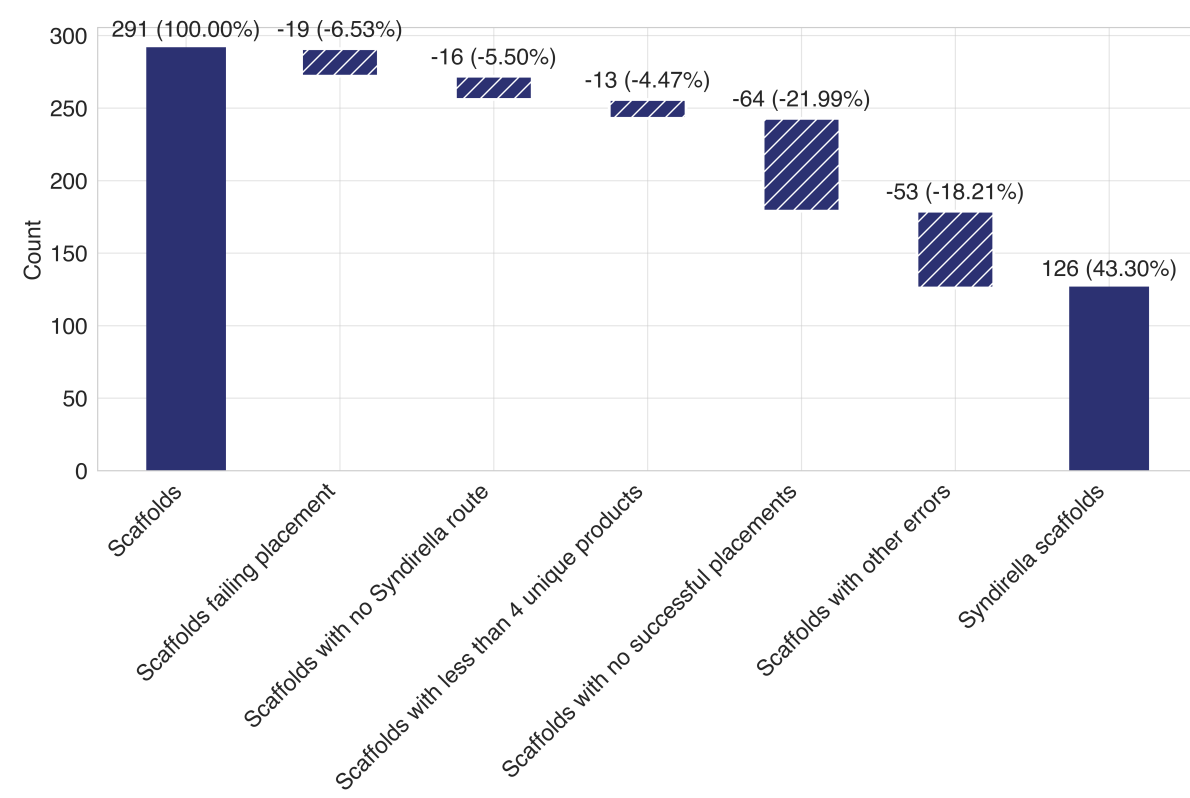

(d)

Figure S1: Waterfall plots showing the attrition of scaffolds at different pipeline points for (a) all targets, (b) CHIKV-Mac, (c) EV-A71-2A, and (d) EV-D68-3C-PROA. The absolute values are noted above each bar either by noting the loss (for hatched bars) or absolute value (for solid bars). The fraction of each value to the input is in parentheses.

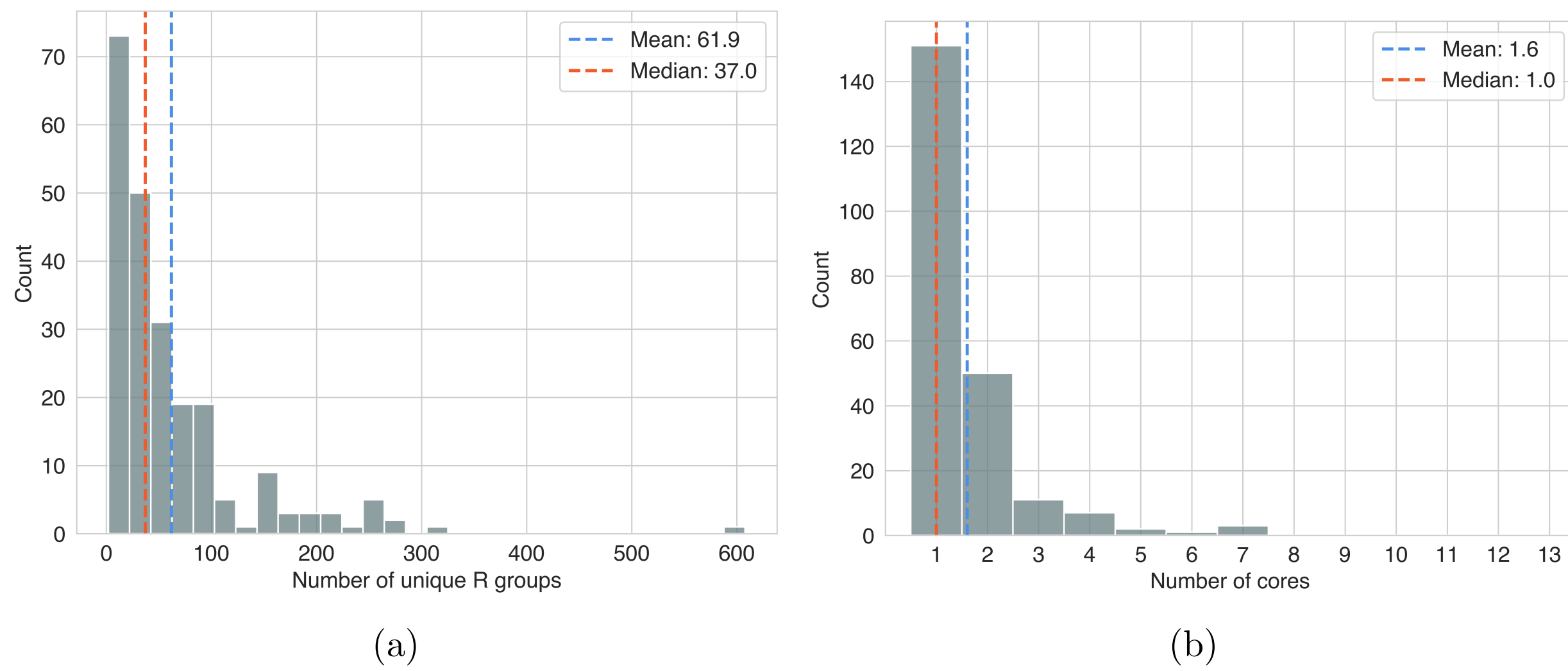

Figure S2: Syndirella produces series with unique R groups and more than one core for the successful-234. (a) Histogram of the number unique R groups added per series. (b) Histogram of the number of cores found in each series.

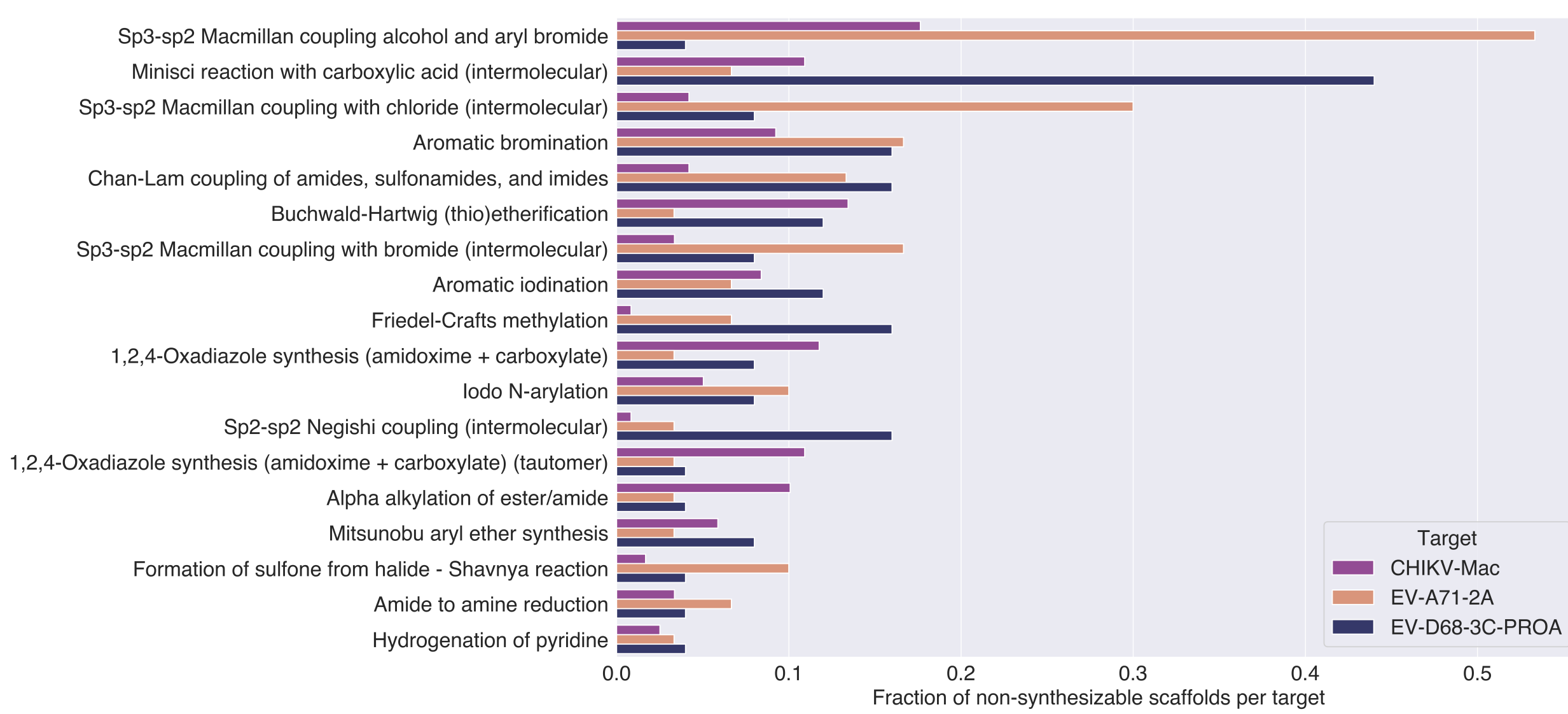

Figure S3: Bar chart of the fraction of non-synthesizable scaffolds per target with reaction in the top five routes returned by Manifold. The reactions shown are the 18 found in the consensus for all targets and their scaffolds with no routes that contained Syndirella encoded reactions.

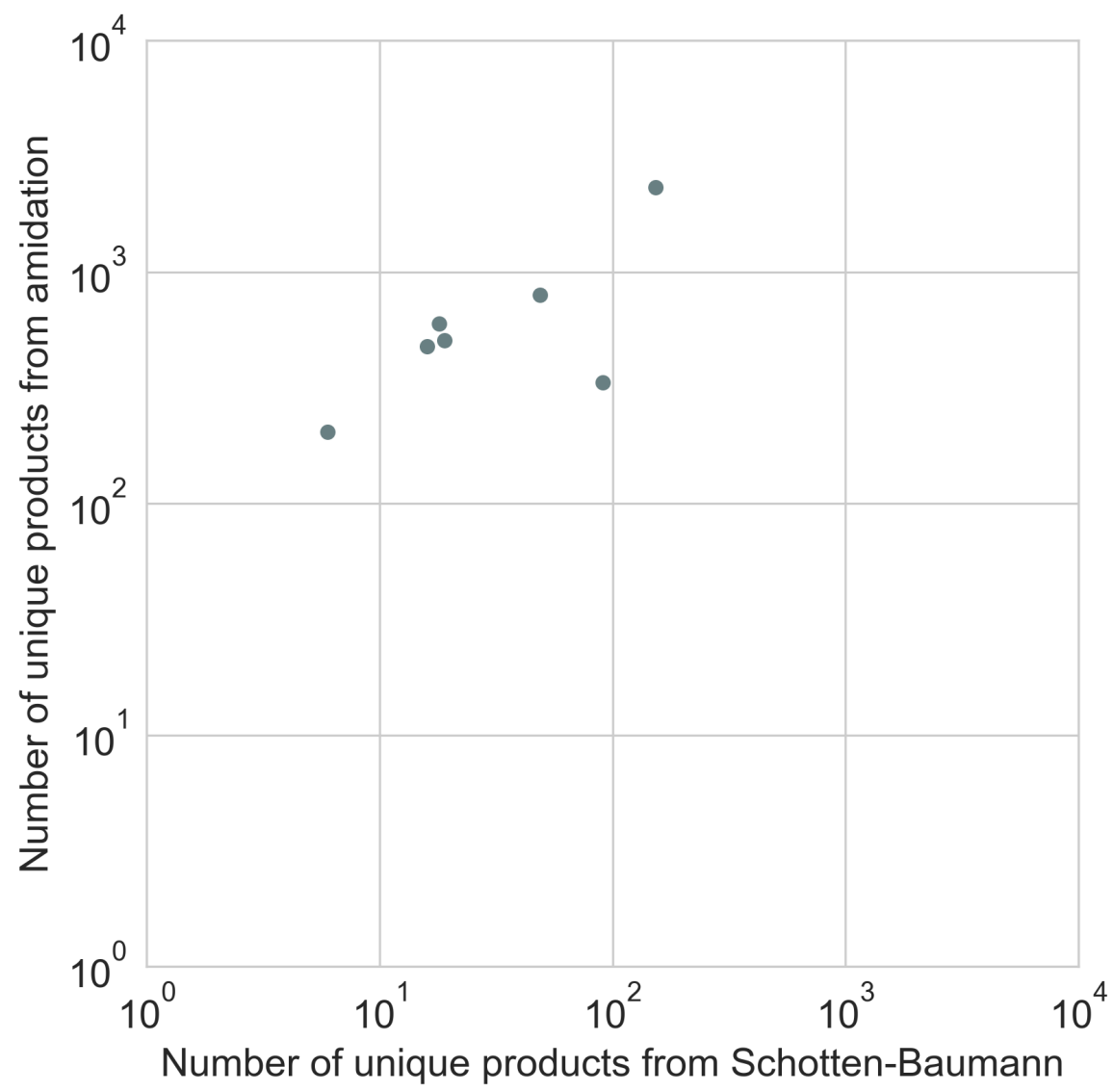

Figure S4: More elaborations are found using amidation than Schotten-Baumann for the same scaffolds. Scatterplot of the number of unique compounds found from Schotten-Baumann versus amidation for N=7 scaffolds.

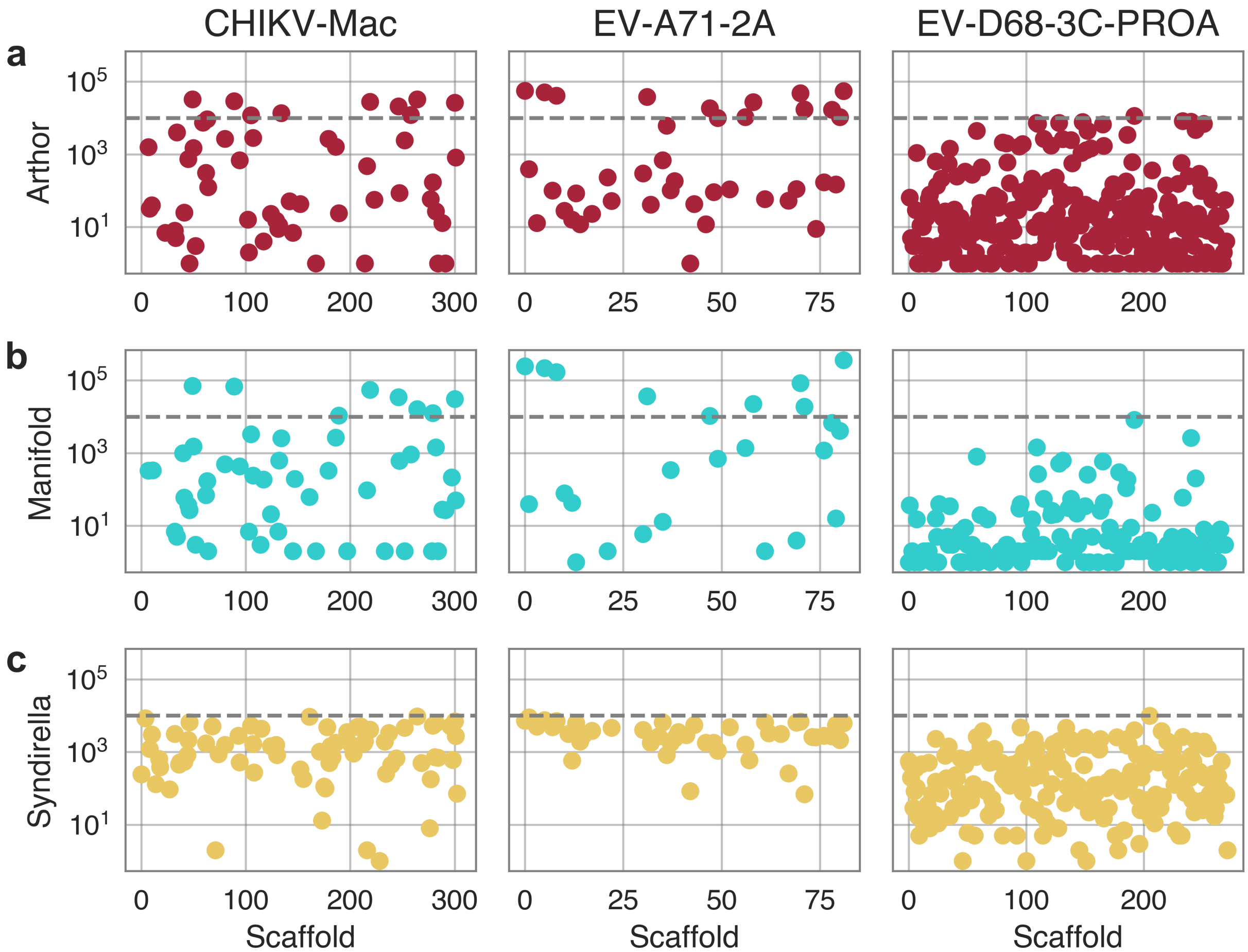

Figure S5: Syndirella has a better tradeoff between breadth and depth for a superstructure search compared to Arthor and Manifold which have a greater depth generally for fewer scaffolds. **a** compounds found per target by Arthor, **b** by Manifold, and **c** by Syndirella.

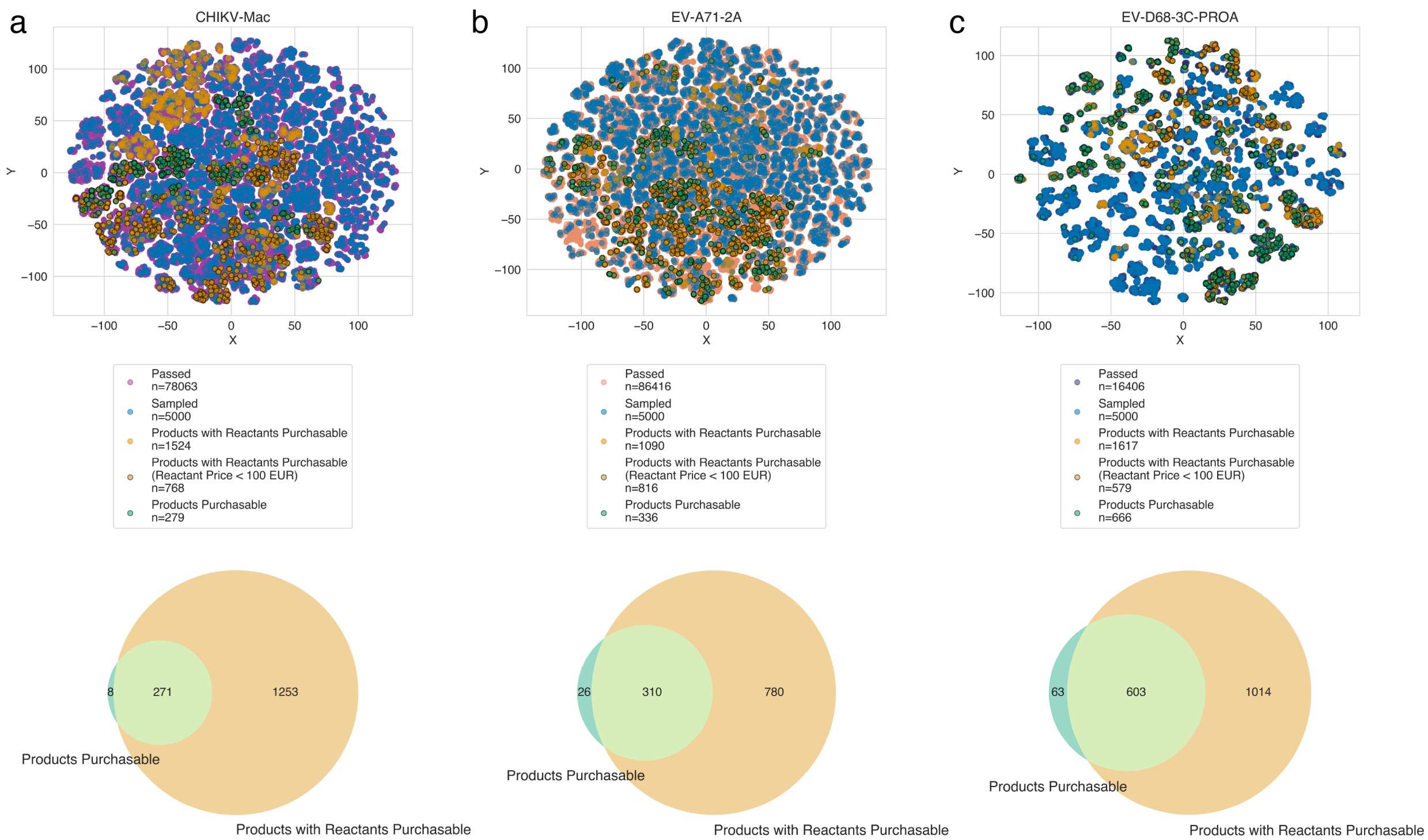

Figure S6: Chemical space of the sampled and accessibility status by either reactant purchasable or product purchasable method in Enamine. **a** chemical space projected onto two dimensions using the t-SNE algorithm colored by purchase status in Enamine and the Venn diagram of the overlapping compounds for CHIKV-Mac, **b** EV-A71-2A, and **c** EV-D68-3C-PROA.

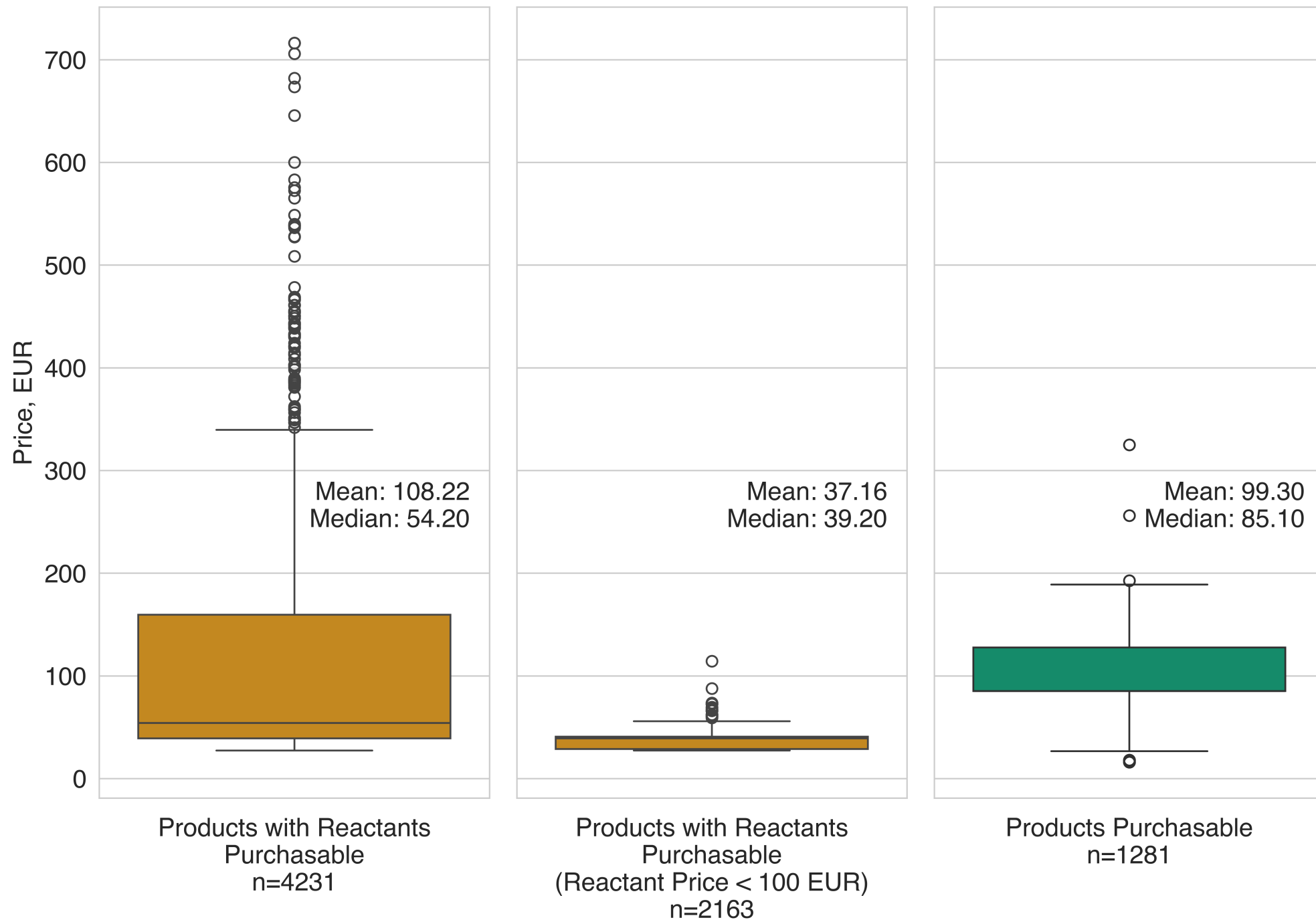

Figure S7: On average, the price per product by purchasing reactants is cheaper than purchasing the product directly when restricting to <100 EUR price per reactant. Box plots of the distribution of prices after Enamine quoting either by summing the reactant prices for a product (left), only including products with reactant price <100 EUR (center), and direct product price (right).

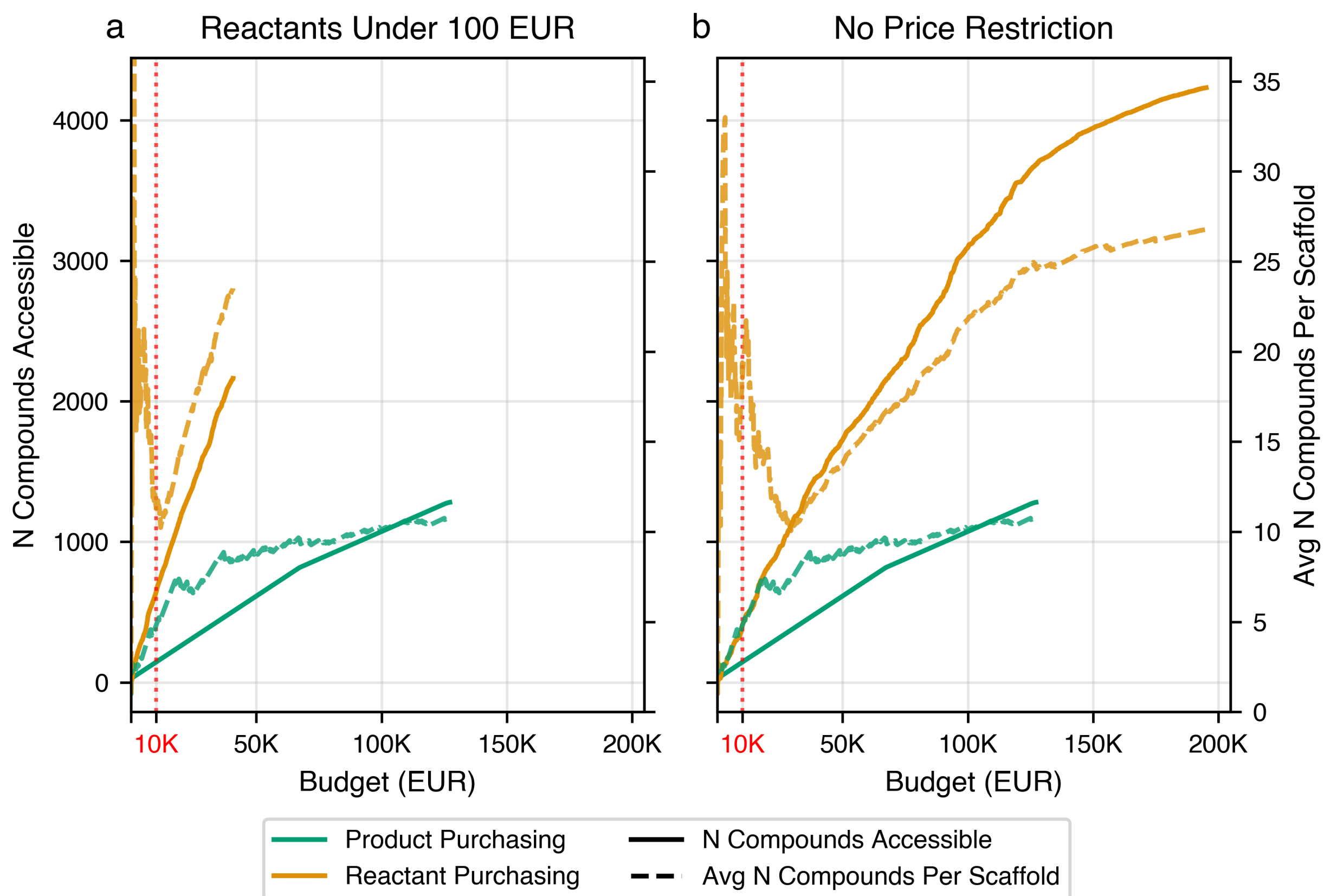

Figure S8: For a given budget, more compounds are accessible by purchasing reactants rather than purchasing the products directly, even when including experimental synthesis costs. Budget and number of compounds accessible either by product purchasing (green) or reactant purchasing (gold). Compounds are ordered by ascending price of the lowest reactant price for reactant purchasing or product price for product purchasing. For reactant purchasing, the total cost includes both reagent procurement and synthesis expenses, calculated as the sum of reactant prices plus 6.11 EUR per compound synthesis cost (Table S4). **a** Compounds accessible where all reactants have individual prices $<$ 100 EUR. **b** Compounds accessible with no restriction. For each subplot a vertical red line at 10K EUR cumulative budget is added.

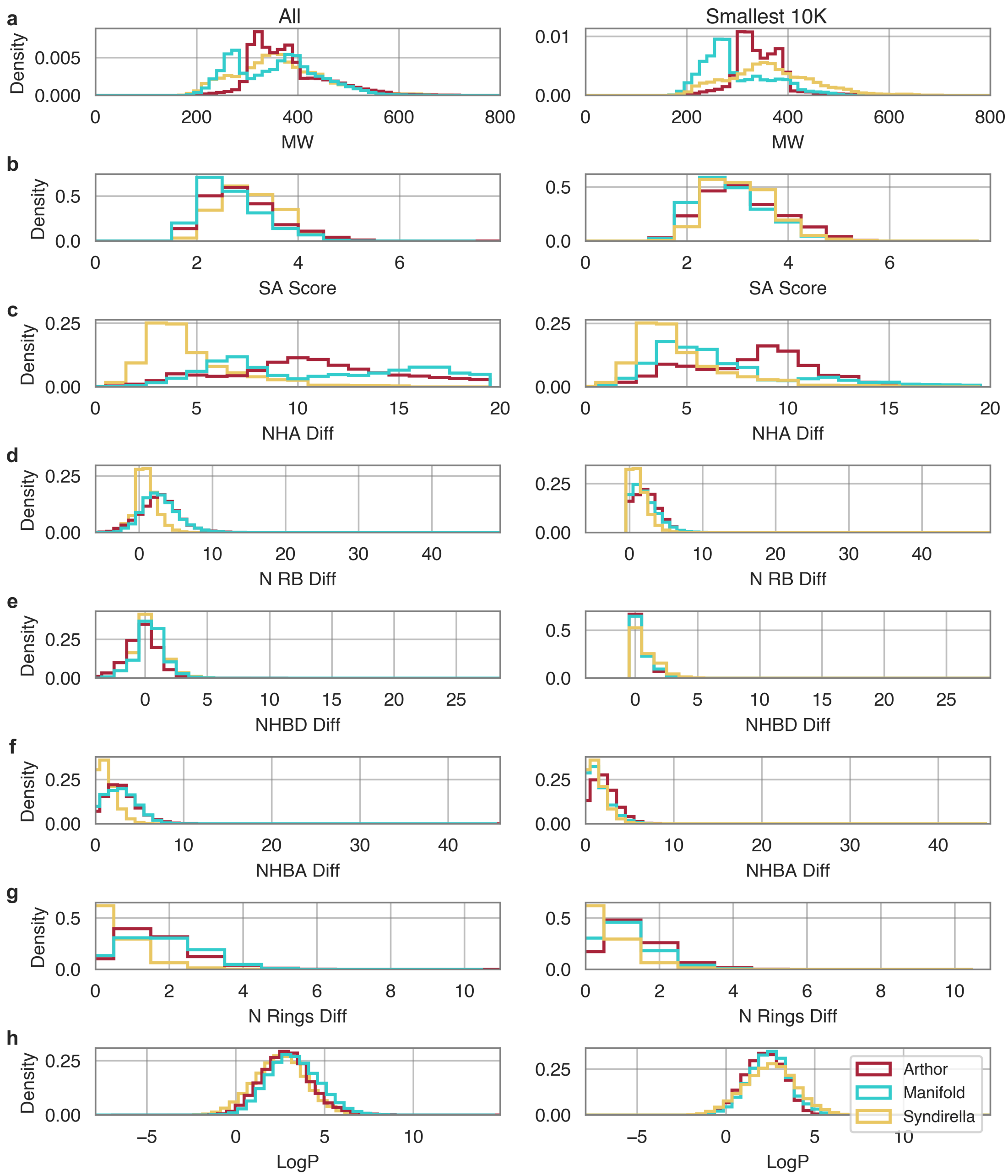

Figure S9: Property distributions of compounds found by superstructure methods either with no restriction (left column) or restricted to the top 10K compounds ordered by ascending molecular weight (right column). **a** molecular weight (MW), **b** synthetic accessibility (SA) score calculated by RDKit, **c** number of heavy atoms difference (NHA Diff) to the scaffold, **d** number of rotatable bonds difference (N RB Diff) to the scaffold, **e** number of hydrogen bond donors difference (NHBD Diff) to the scaffold, **f** number of hydrogen bond acceptors difference (NHBA Diff) to the scaffold, **g** number of rings different to the scaffold, **h** LogP calculated using RDKit's Crippen method. For all distributions the x-axis is limited to the largest 99.9999 percentile value of the three methods.

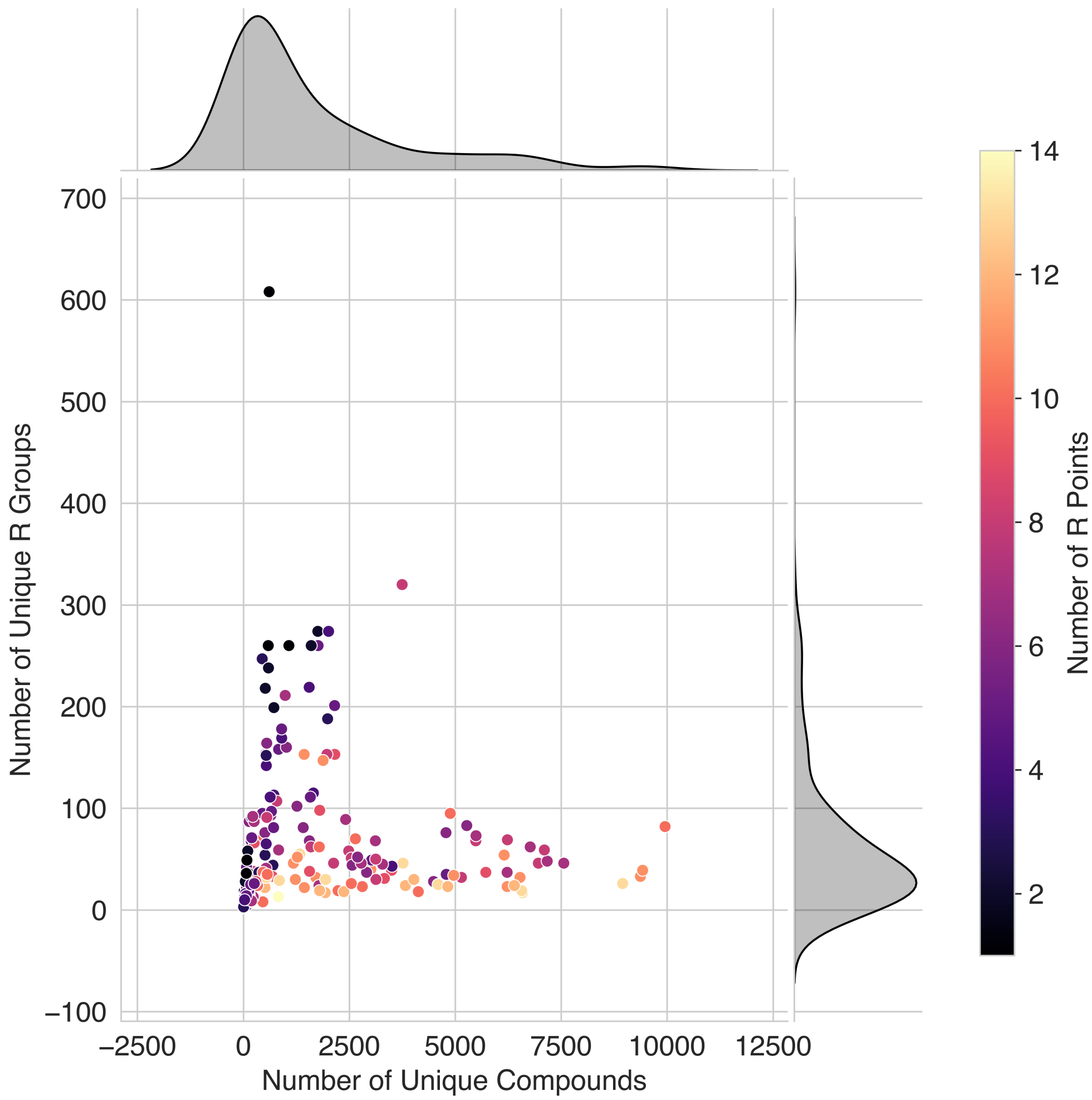

Figure S10: Scatter plot of the number of unique compounds and the number of unique R groups colored by number of R points on the scaffold per target.

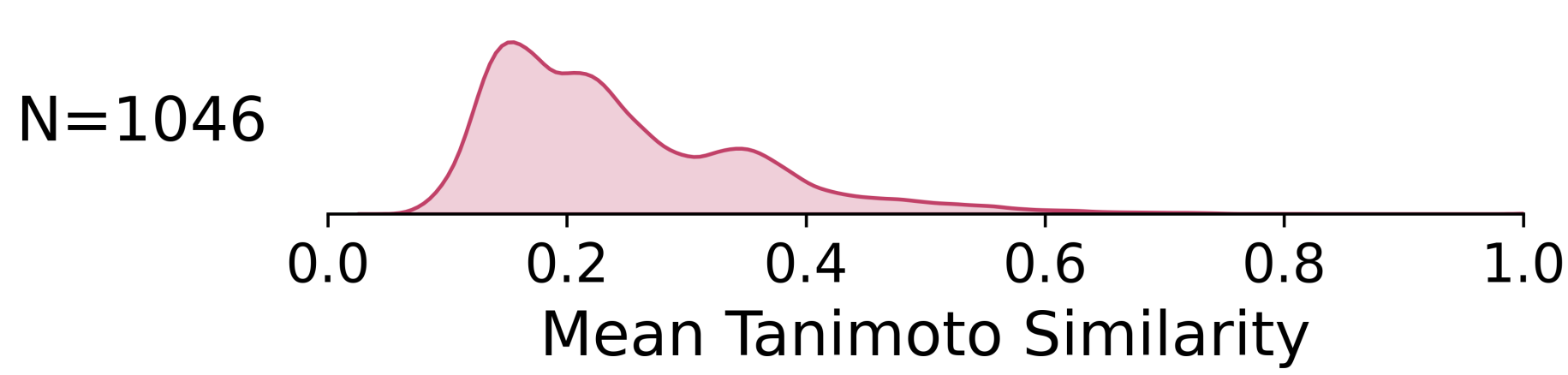

Figure S11: Average tanimoto similarity kernel density estimates for all compounds made in the 3-amino-pyridine-like series for the COVID Moonshot between 2020-03-17 to 2021-06-01 and limited to 10 new atoms added to the TRY-UNI-714a760b-6 scaffold.

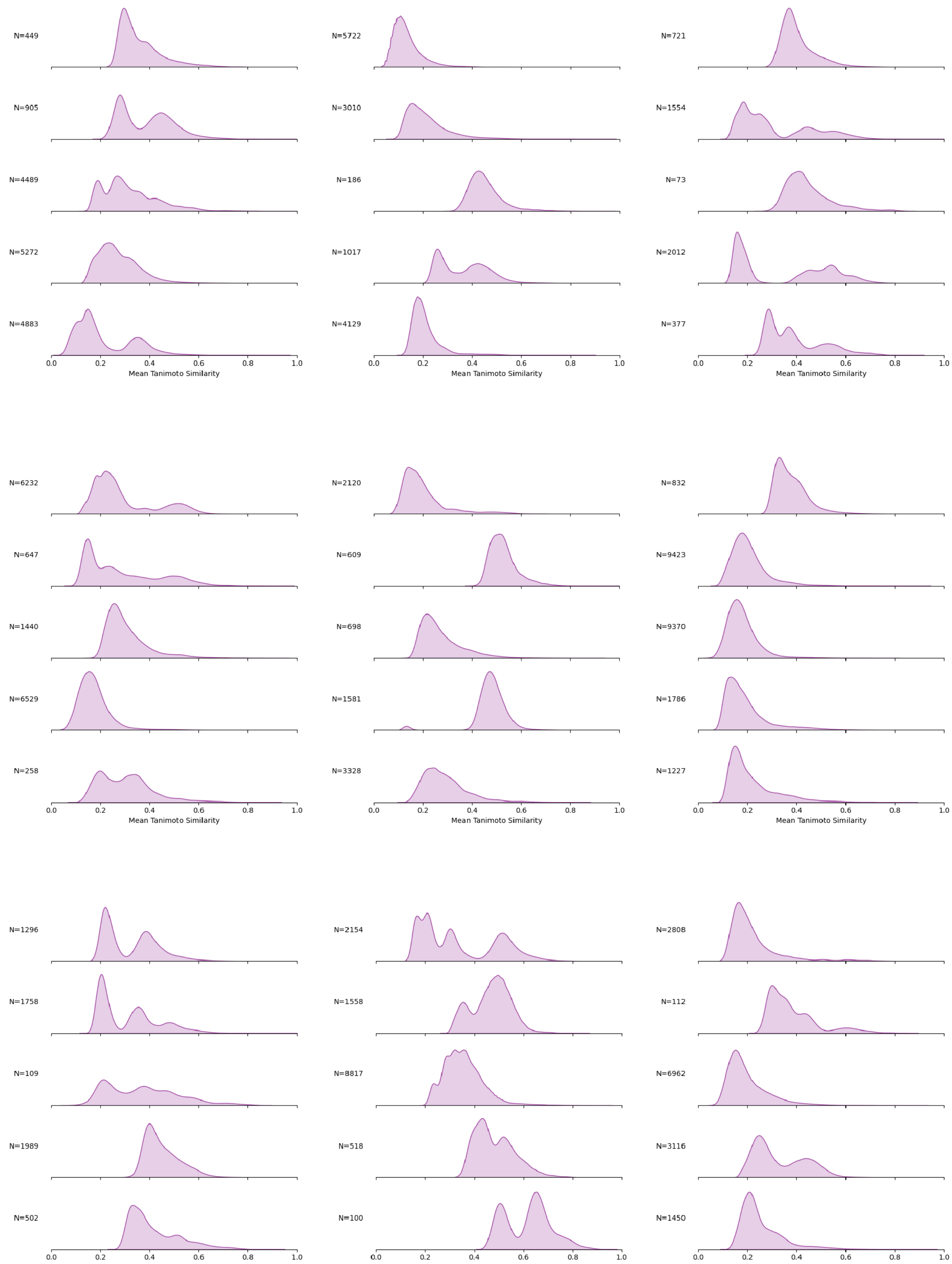

Figure S12: Set 1 of 2 of CHIKV-Mac average tanimoto similarity kernel density estimates per scaffold cluster.

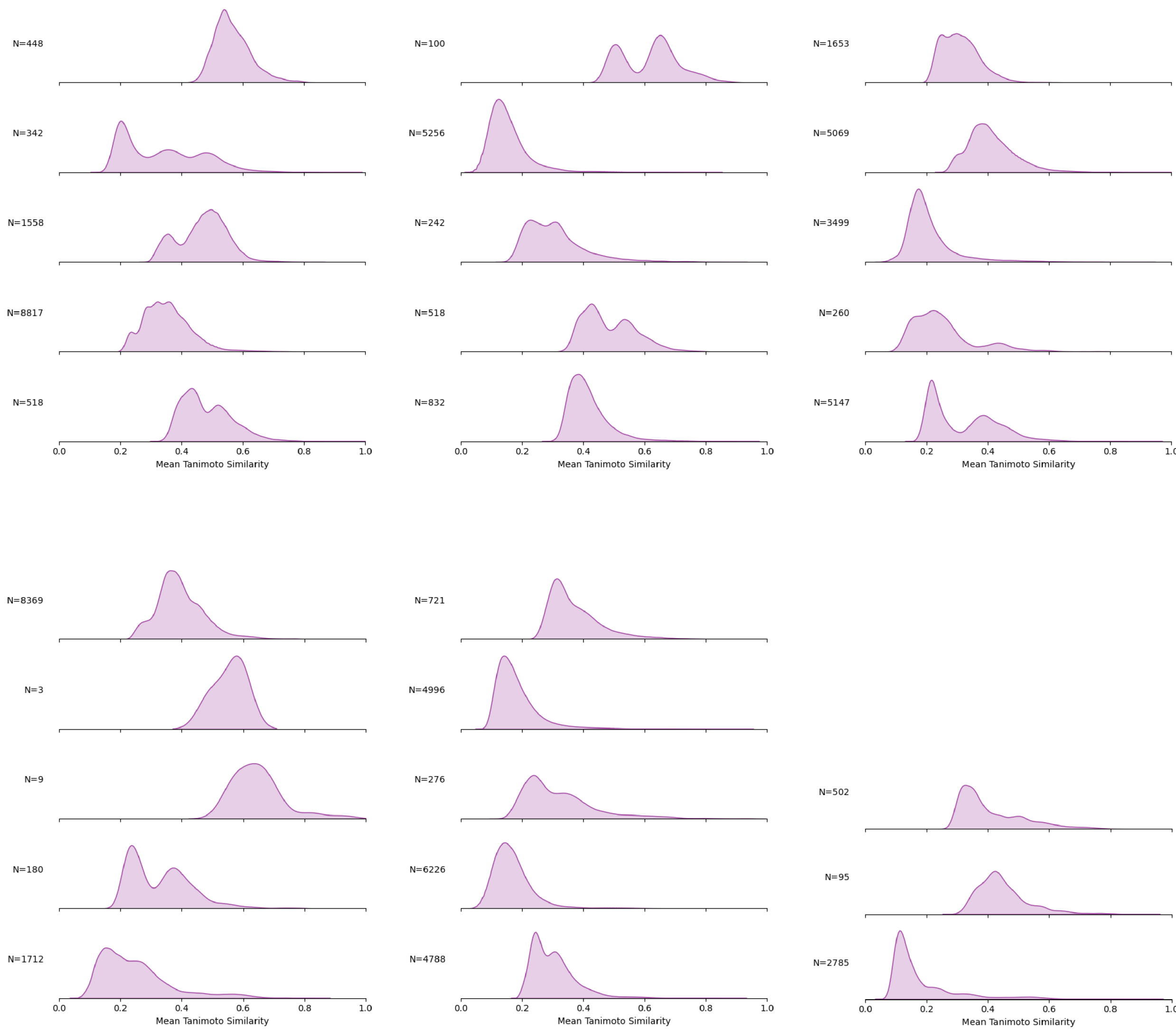

Figure S13: Set 2 of 2 of CHIKV-Mac average tanimoto similarity kernel density estimates per scaffold cluster.

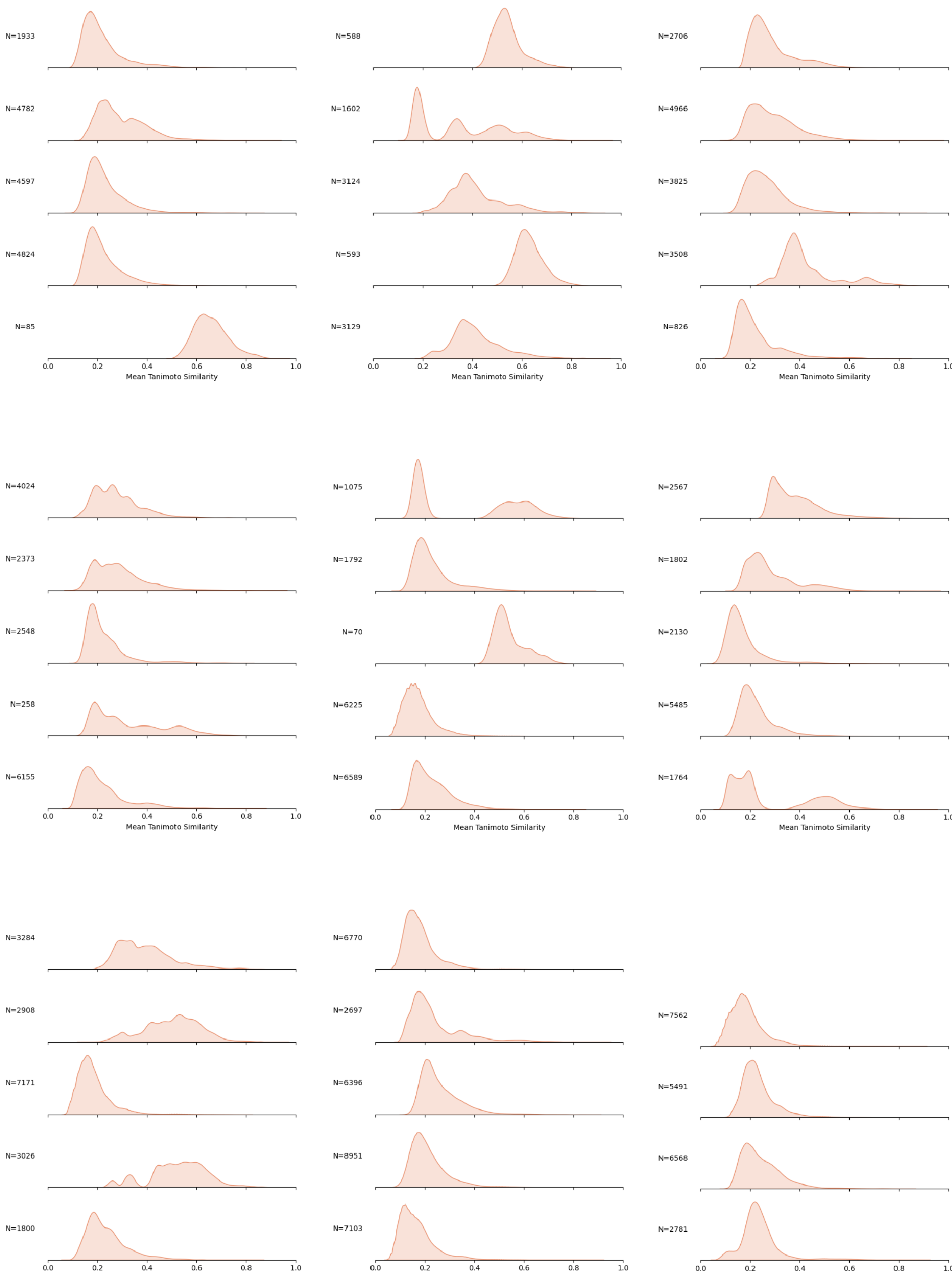

Figure S14: Set 1 of 1 of EV-A71-2A average tanimoto similarity kernel density estimates per scaffold cluster.

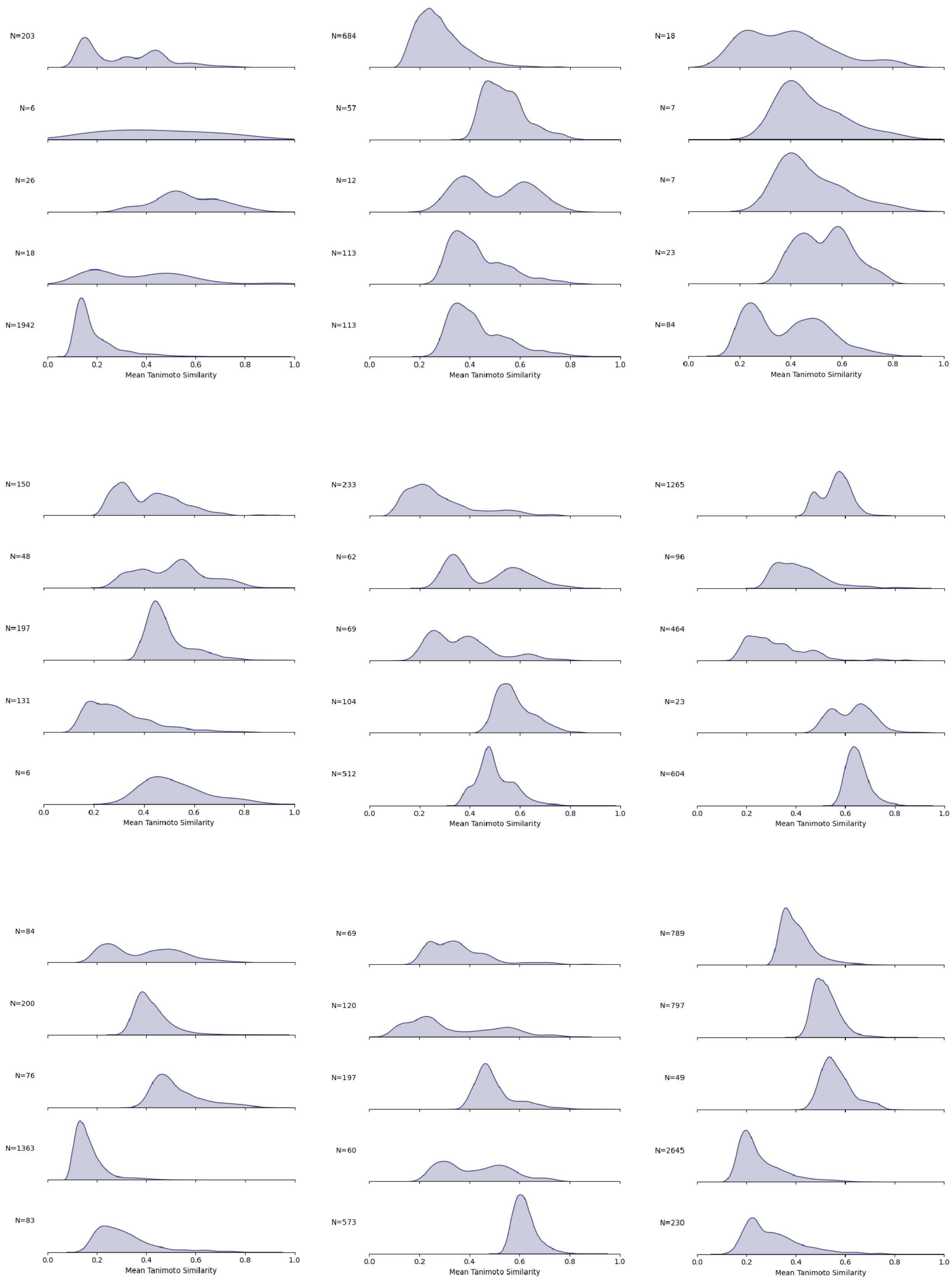

Figure S15: Set 1 of 5 of EV-D68-3C average tanimoto similarity kernel density estimates per scaffold cluster.

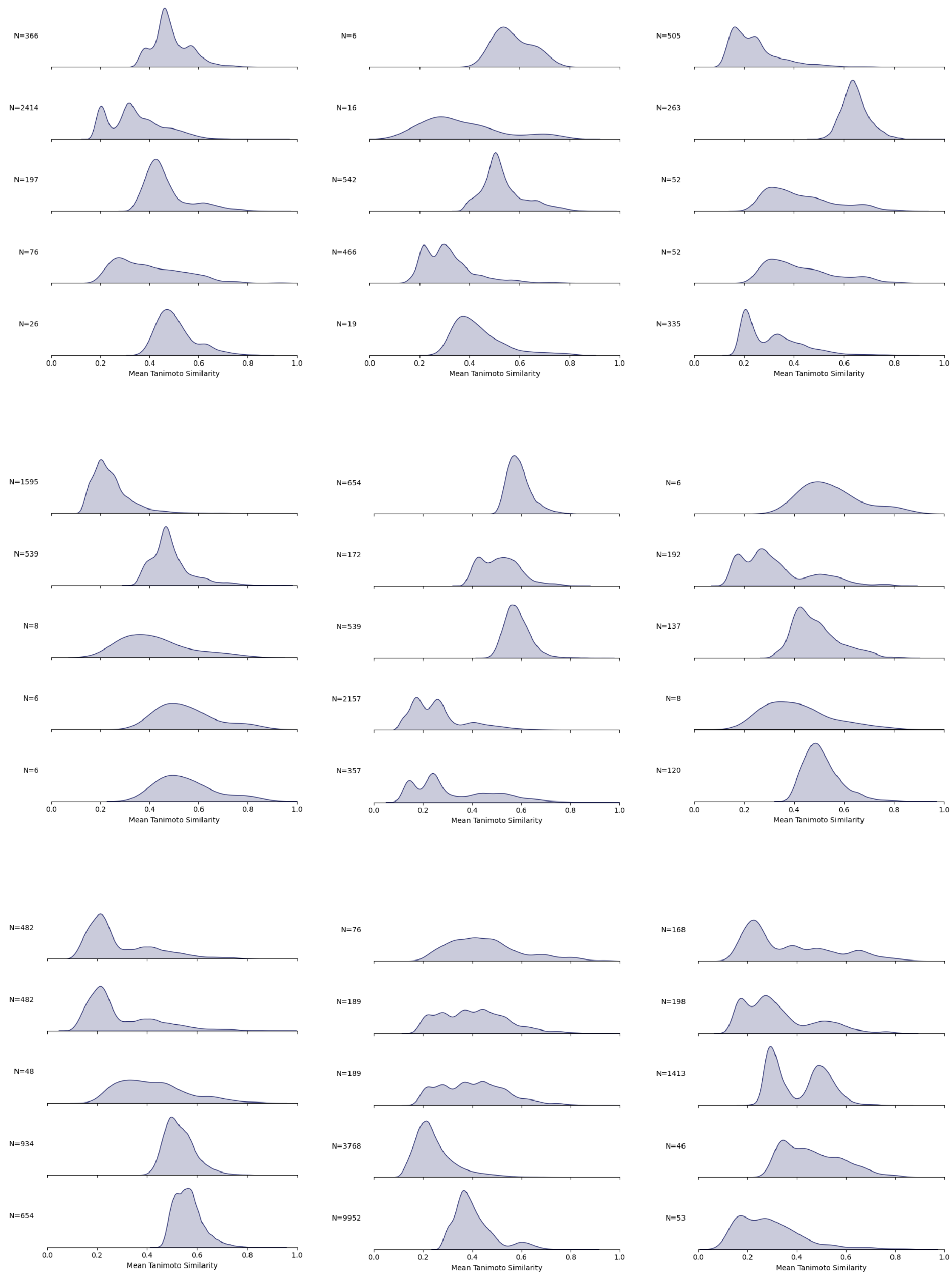

Figure S16: Set 2 of 5 of EV-D68-3C average tanimoto similarity kernel density estimates per scaffold cluster.

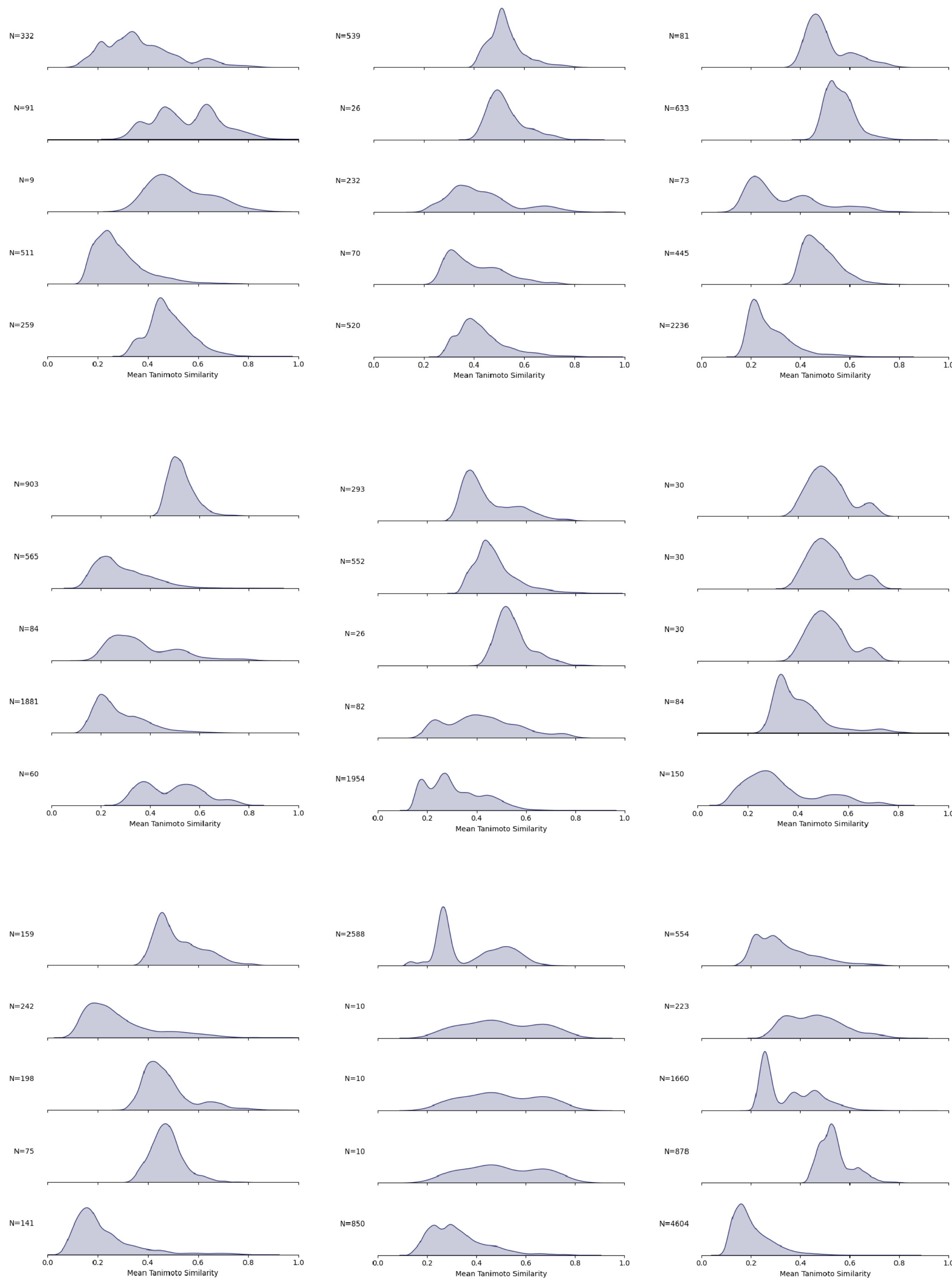

Figure S17: Set 3 of 5 of EV-D68-3C average tanimoto similarity kernel density estimates per scaffold cluster.

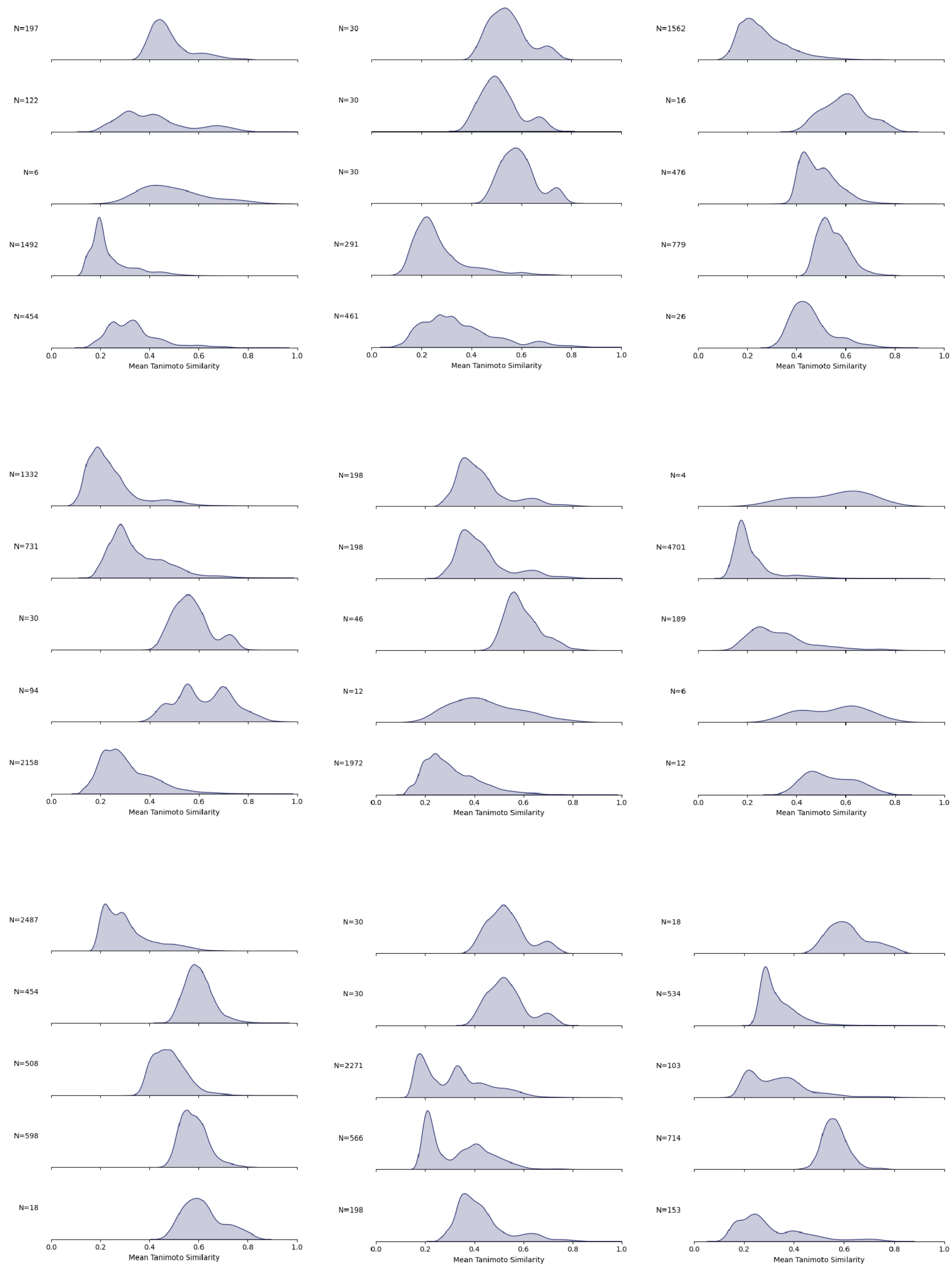

Figure S18: Set 4 of 5 of EV-D68-3C average tanimoto similarity kernel density estimates per scaffold cluster.

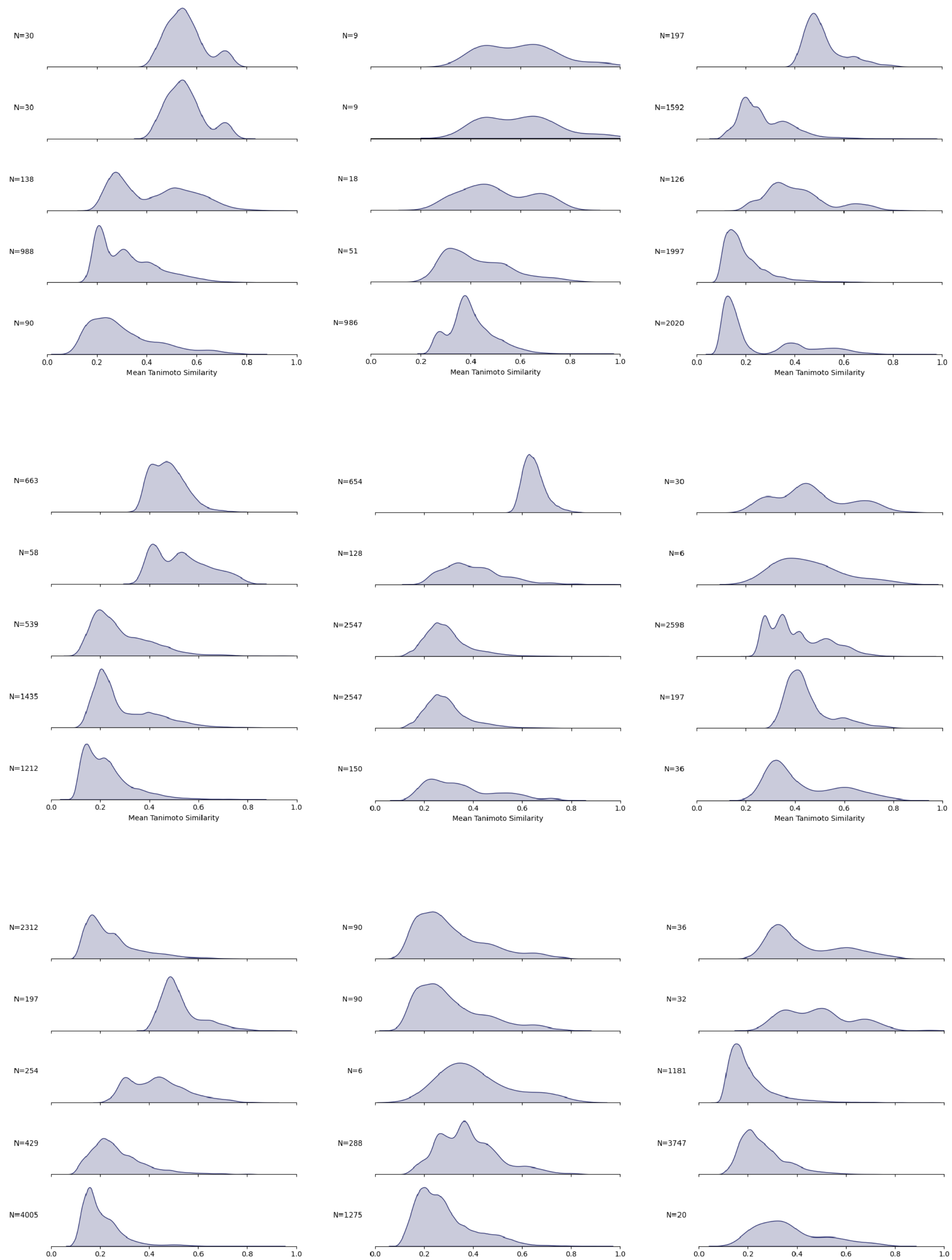

Figure S19: Set 5 of 5 of EV-D68-3C average tanimoto similarity kernel density estimates per scaffold cluster.

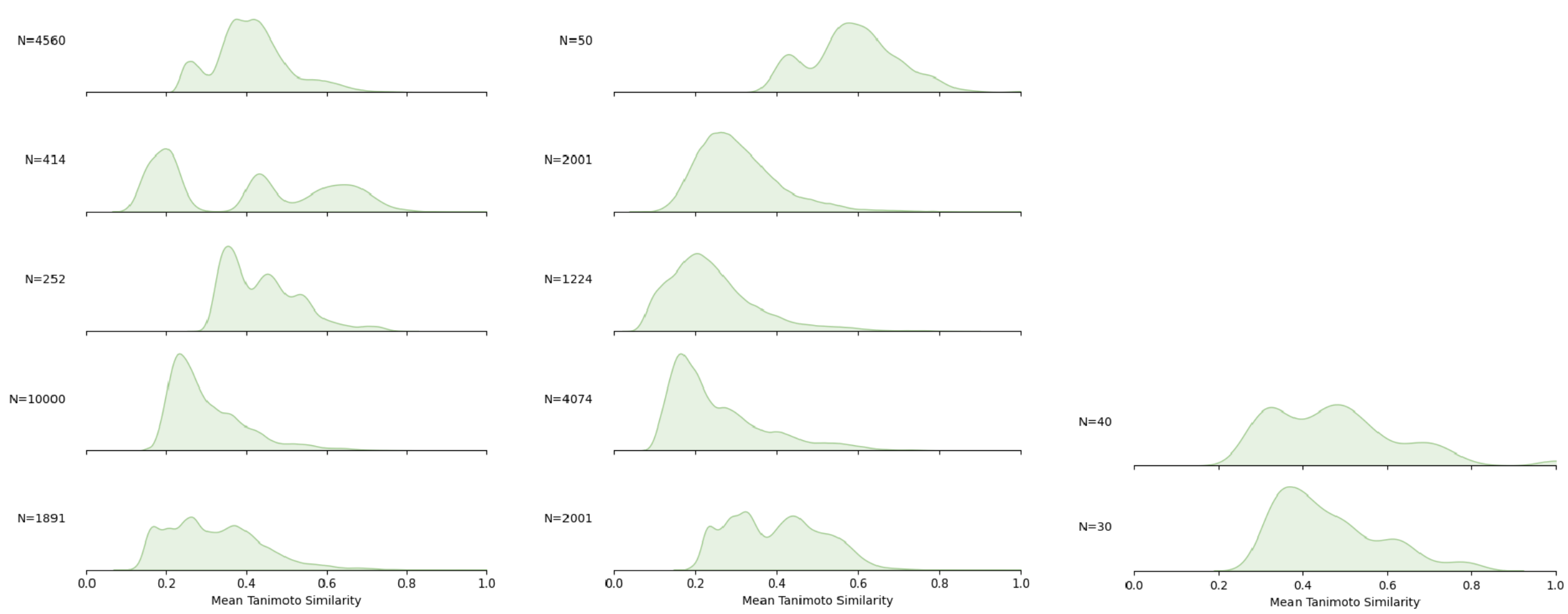

Figure S20: NCS1 average tanimoto similarity kernel density estimates per scaffold cluster.
