# Supplementary material for: Syndirella: synthesis-directed fragment elaboration enables extensive and cost-effective binding site exploration beyond catalog compounds
Source: Chem Sci. 2026 Sep 29;17(41):20118–39. doi: 10.1039/d6sc02032a (PMC13656017; doi:10.1039/d6sc02032a)
Supplement: Supplementary file 2 [file SC-017-D6SC02032A-s002.zip › SI/CHIKV_interaction_punchcard_4_updated_edited.pdf]

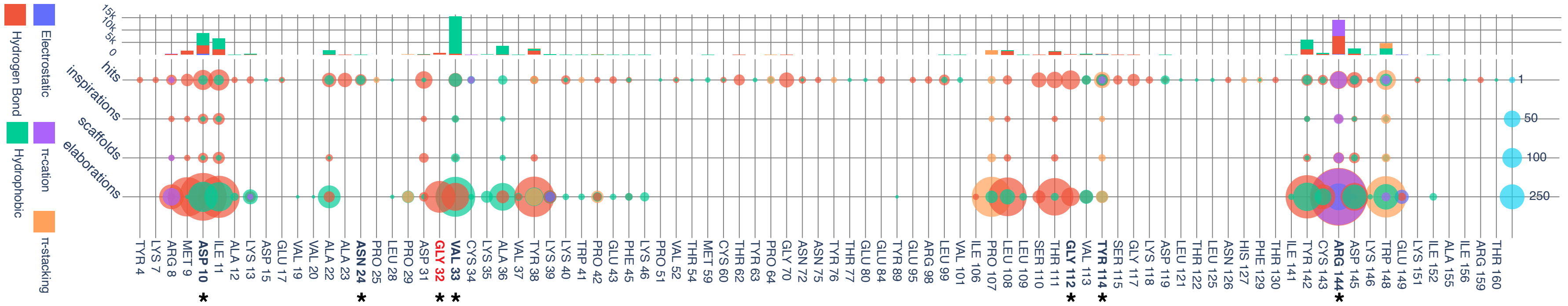
Hydrogen Bond
Electrostatic
Hydrophobic
π-cation
π-stacking
15k
10k
5k
0
hits
inspirations
scaffolds
elaborations
1
50
100
250
TYR 4
LYS 7
ARG 8
MET 9
ASP 10
ILE 11
ALA 12
LYS 13
ASP 15
GLU 17
VAL 19
VAL 20
ALA 22
ALA 23
ASN 24
PRO 25
LEU 28
PRO 29
ASP 31
GLY 32
VAL 33
CYS 34
LYS 35
ALA 36
VAL 37
TYR 38
LYS 39
LYS 40
TRP 41
PRO 42
GLU 43
PHE 45
LYS 46
PRO 51
VAL 52
THR 54
MET 59
CYS 60
THR 62
TYR 63
PRO 64
GLY 70
ASN 72
ASN 75
TYR 76
THR 77
GLU 80
GLU 84
TYR 89
GLU 95
ARG 98
LEU 99
VAL 101
ILE 106
PRO 107
LEU 108
LEU 109
SER 110
THR 111
GLY 112
VAL 113
TYR 114
SER 115
GLY 117
LYS 118
ASP 119
LEU 121
THR 122
LEU 125
ASN 126
HIS 127
PHE 129
THR 130
ILE 141
TYR 142
CYS 143
ARG 144
ASP 145
LYS 146
TRP 148
GLU 149
LYS 151
ILE 152
ALA 155
ILE 156
ARG 159
THR 160
*
*
*
*
*
*
*
